# Evolution of the “Internet Plus Health Care” Mode Enabled by Artificial Intelligence: Development and Application of an Outpatient Triage System

**DOI:** 10.2196/51711

**Published:** 2024-10-30

**Authors:** Lingrui Yang, Jiali Pang, Song Zuo, Jian Xu, Wei Jin, Feng Zuo, Kui Xue, Zhongzhou Xiao, Xinwei Peng, Jie Xu, Xiaofan Zhang, Ruiyao Chen, Shuqing Luo, Shaoting Zhang, Xin Sun

**Affiliations:** 1 Clinical Research and Innovation Unit Xinhua Hospital Affiliated to Shanghai Jiao Tong University School of Medicine Shanghai China; 2 Shanghai Artificial Intelligence Laboratory Shanghai China

**Keywords:** artificial intelligence, triage system, all department recommendation, subspecialty department recommendation, “internet plus healthcare”, “internet plus health care”

## Abstract

**Background:**

Although new technologies have increased the efficiency and convenience of medical care, patients still struggle to identify specialized outpatient departments in Chinese tertiary hospitals due to a lack of medical knowledge.

**Objective:**

The objective of our study was to develop a precise and subdividable outpatient triage system to improve the experiences and convenience of patient care.

**Methods:**

We collected 395,790 electronic medical records (EMRs) and 500 medical dialogue groups. The EMRs were divided into 3 data sets to design and train the triage model (n=387,876, 98%) and test (n=3957, 1%) and validate (n=3957, 1%) it. The triage system was altered based on the current BERT (Bidirectional Encoder Representations from Transformers) framework and evaluated by recommendation accuracies in Xinhua Hospital using the cancellation rates in 2021 and 2022, from October 29 to December 5. Finally, a prospective observational study containing 306 samples was conducted to compare the system’s performance with that of triage nurses, which was evaluated by calculating precision, accuracy, recall of the top 3 recommended departments (recall@3), and time consumption.

**Results:**

With 3957 (1%) records each, the testing and validation data sets achieved an accuracy of 0.8945 and 0.8941, respectively. Implemented in Xinhua Hospital, our triage system could accurately recommend 79 subspecialty departments and reduce the number of registration cancellations from 16,037 (3.83%) of the total 418,714 to 15,338 (3.53%) of the total 434200 (*P*<.05). In comparison to the triage system, the performance of the triage nurses was more accurate (0.9803 vs 0.9153) and precise (0.9213 vs 0.9049) since the system could identify subspecialty departments, whereas triage nurses or even general physicians can only recommend main departments. In addition, our triage system significantly outperformed triage nurses in recall@3 (0.6230 vs 0.5266; *P*<.001) and time consumption (10.11 vs 14.33 seconds; *P*<.001).

**Conclusions:**

The triage system demonstrates high accuracy in outpatient triage of all departments and excels in subspecialty department recommendations, which could decrease the cancellation rate and time consumption. It also improves the efficiency and convenience of clinical care to fulfill better the usage of medical resources, expand hospital effectiveness, and improve patient satisfaction in Chinese tertiary hospitals.

## Introduction

Recent advancements in technology, including the internet, the internet of things, artificial intelligence (AI), 5G, big data, and other emerging technologies, have made it possible to process medical data in order to enhance the effectiveness and convenience of patient treatment [1]. The emergence of “internet plus health care” allows online prehospital registration, diagnosis, and treatment [2-5]. Despite all the breakthroughs in health care technologies, billions of patients flock into tertiary hospitals for medical consultations every year, enduring the “3 long, 1 short” problem in China [6]. Using current online triage systems to help these patients during online registrations is not enough. As current triage systems in China mainly rely on manually maintained department templates, inaccurate department recommendations may occur when the input exceeds their scope or revisions are needed. Meanwhile, patients still struggle to choose the right department due to a lack of medical knowledge and the various specialist outpatient departments in tertiary hospitals [7]. Triage errors can waste time and medical resources, cause unpleasant medical experiences, and even result in misdiagnosis and treatment delays [8,9].

The universal usage of electronic medical records (EMRs) and AI has enabled the processing of medical data for better patient triage before clinical care, but there are still obstacles when applying AI-based triage systems to Chinese health care institutions [10-15]. As most current triage systems are designed for specialized departments, neglecting the numerous subspecialty departments in hospitals, achieving all-department recommendations remains a main challenge because Chinese patients do not rely on referrals before registrations [16]. Another significant challenge is using Chinese-based patient complaints as triage system inputs. Variations in word segmentations between English and Chinese [17,18] and the variability of patient descriptions significantly contribute to this challenge [19,20]. For example, “I feel pain in my head” could also be described as “headache.”

To further improve the “internet plus health care” mode and achieve accurate, efficient, and convenient medical services in China with better usage of clinical resources, this study proposed a precise triage system for Chinese tertiary hospitals that leverages patient-entered chief complaints as input to provide all-department recommendations.

## Methods

### Study Design

In this study, we designed an AI-based triage system for all-department triage in Chinese tertiary hospitals. All the clinical data were collected from Xinhua Hospital affiliated with the Shanghai Jiao Tong University School of Medicine, a comprehensive tertiary hospital that played a leading role throughout the process of medical digitization in China. We collected 500 medical dialogue groups from an online platform called Spring Rain and used them to design and train the triage system. After implementation of the system, we validated its performance by estimating cancellation rates and conducting a prospective observational study to compare the triage system with experienced triage nurses.

### Construction of the Triage System

**Figure 1:**
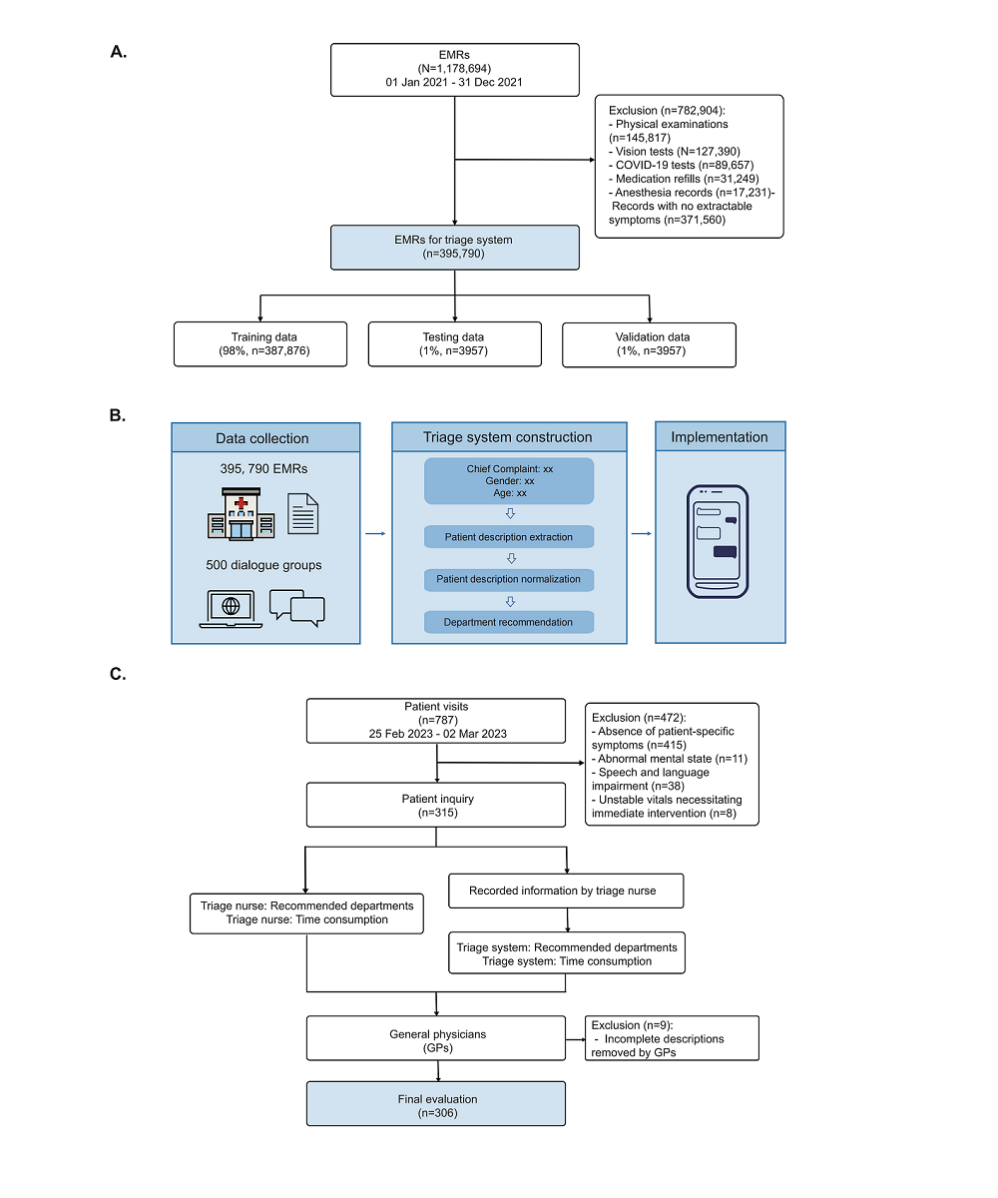
Study overview of data processing for model training, the model framework, and the study design to compare the triage system with triage nurses: (A) processing of EMRs, (B) construction of the triage system and implementations, and (C) workflow of comparing the triage system with triage nurses. EMR: electronic medical record; GP: general physician.

For the development of the triage system, we conducted a retrospective study and obtained EMRs from Xinhua Hospital, which encompasses 79 departments and specializes in pediatric diseases, with 1,178,694 outpatient visits from January 1 to December 31, 2021. A total of 395,790 (33.58%) EMRs, including chief complaints, present medical histories, and past medical histories, were selected after excluding records that contained physical examinations (n=145,817, 12.37%), vision tests (n=127,390, 10.81%), COVID-19 tests (n=89,657, 7.61%), medication refills (n=31,249, 2.65%), anesthesia records (n=17,231, 1.46%), and records with no extractable symptoms (n=371,560, 31.52%), as shown in Figure 1A. Meanwhile, 500 spoken dialogue groups were collected to extract patients’ oral expressions of symptoms. Sensitive information was removed, and EMRs were deidentified.

Our model was altered based on the current BERT (Bidirectional Encoder Representations from Transformers) [21] framework to enhance model adaptability to Chinese oral expressions. The construction of the triage system can be summarized in 3 steps (Figure 1B): First, a patient description extraction module was designed to extract entities (vital medical events), such as symptoms, anatomical location, and existence from patient descriptions (Multimedia Appendix 1). Second, a patient description normalization module was designed to convert nonstandard symptoms and anatomical locations into standardized medical terminologies according to the International Classification of Diseases, Injuries and Causes of Death*, 10th Edition* (ICD-10), the most official code for diseases, symptoms, and anatomical locations acceptable for doctors, which eliminates uncertainty in patient oral expressions (Multimedia Appendix 1). Third, a department recommendation module was designed to recommend departments for patients based on standardized medical concepts, patients’ age, and patients’ gender. (Multimedia Appendix 1).

### Model Training and Validation

We developed 2 strategies to train the model: first, training the model with all EMRs (original) and then training the model with downsampled chief complaints from EMRs to simulate patient complaints. Chief complaints in EMRs included more symptoms and were more comprehensive than patient descriptions. Specifically, the median number of symptoms in EMRs was 4 (IQR 2-7), while the median number of symptoms in patient oral expressions was 1 (IQR 0-1), as shown in Multimedia Appendix 2. Thus, for each chief complaint in the EMRs, we randomly selected symptoms to reduce symptoms contained in the EMRs. Specifically, for a sentence in chief complaints, if the existence of a certain extracted symptom was “yes,” the probability of sampling it was 50%, otherwise the probability of sampling it was 20% (Multimedia Appendix 3). We split the original and downsampled EMRs into training data (n=387,876, 98%), testing data (n=3957, 1%), and validation data (n=3957, 1%). The 500 dialogue groups were split accordingly and put into the model training stage (patient description extraction module) so that the model could understand the difference between medical terminologies and patient chief complaints to further normalize patient descriptions. Next, we evaluated the performance of our triage system by testing the 2 training strategies on both the original and downsampled testing and validation data sets. We calculated the accuracy of the top 1 and 3 recommended departments to evaluate department recommendation. Precision, recall, and the *F*_1_-score were calculated to evaluate the patient description extraction module (Multimedia Appendix 1). These analyses were conducted using Python 3.8.

### System Performance Validated by Cancellation Rate

The triage system was implemented as a mini program on mobile phones at Xinhua Hospital on October 27, 2022. Specifically, the triage system was linked to WeChat through its application programming interface (API), as shown in Multimedia Appendix 1. Records from October 29 to December 5, 2022, were collected to explore the ability of the triage system. The period was chosen to avoid a COVID-19 peak season in Shanghai, which helped reduce bias induced by fever clinics. The cancellation rate of this period was compared with that of the same period in 2021 by a proportional test using R version 4.2.1 (R Foundation for Statistical Computing). We tracked the number of triage system users from January 1 to May 6, 2023, and summarized the trend of user application.

### System Performance Compared With Triage Nurses’ Performance

A prospective observational study containing 306 (38.8%) samples was conducted at Xinhua Hospital from February 25 to March 2, 2023, to compare the triage system’s performance against the triage nurses’ performance. In total, 787 patients presented to the consultation center and 315 (40%) were included. Exclusion criteria included the absence of patient-specific symptoms, an abnormal mental state, speech and language impairment, and unstable vitals necessitating immediate intervention. The sample size was decided through the McNemar test. By setting the type I error at 0.05 and statistical power at 0.9, the required sample size was at least 152 (Multimedia Appendix 4).

During the comparison, departments recommended by 2 different triage nurses and the consultation time were recorded by the staff. The collected symptom information was entered into the triage system to obtain system recommendations and consultation time. The triage nurses had over 3 years of clinical practice, and the recommended departments were limited to 3. Next, 2 general physicians (GPs) with over 10 years of clinical practice evaluated the recommended departments based on the case report. A third GP was consulted when inconsistency occurred. The GPs needed to (1) evaluate whether the first recommended department was precise, (2) identify the recommended departments that made errors, and (3) provide supplementary recommended departments. During this process, 9 records were excluded as the case report was incomplete and 306 records were finally used for evaluation (Figure 1C). All GPs were blinded to the groups. The primary outcome was the precision of the first recommended department, the accuracy of the recommended departments, and recall of the top 3 recommended departments (recall@3) [22]. Accuracy was defined as the proportion of correct departments to recommended departments. Recall@3 was defined as the proportion of correct departments in the top 3 recommendations to all correct recommended departments. The calculation formulas are as follows:



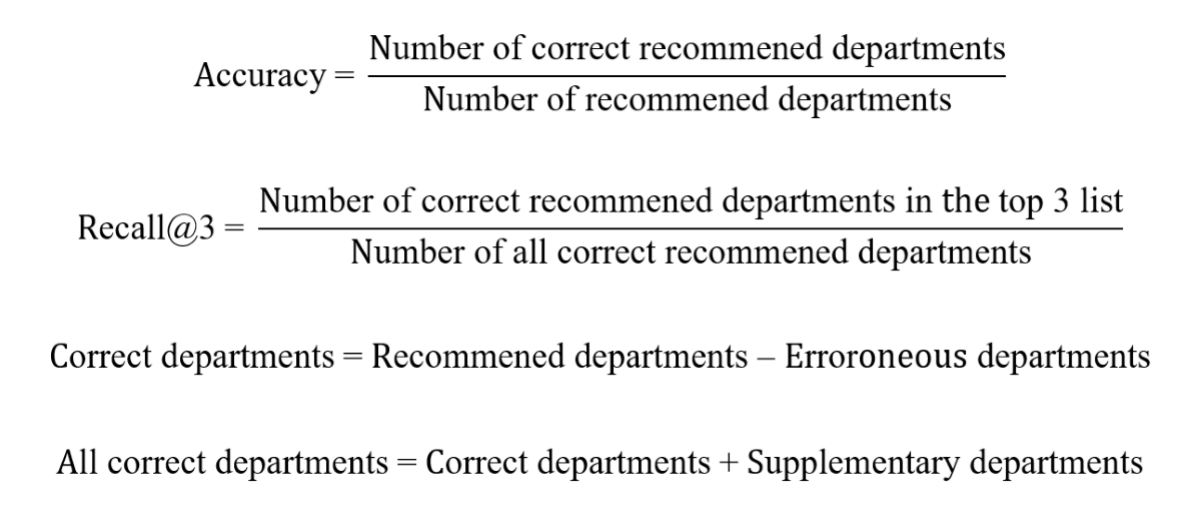



The secondary outcome was the efficiency of the triage process, which was evaluated by the time consumption from the beginning of the triage consultation to receiving the recommended results.

We used the Wilcoxon rank-sum test to test differences in precision, accuracy, recall@3, and time consumption between triage nurses and our triage system. In addition, we conducted a subgroup analysis of adults and children (age<18 years) to evaluate system performance within the 2 subgroups. All analyses were conducted using SAS version 9.4 (SAS Institute) and R version 4.2.1. *P*<.05 was considered statistically significant.

### Ethical Considerations

This retrospective study involved the collection of only basic patient information, such as gender, symptoms, department, diagnosis, and registration status. No patient samples were collected, and no serious adverse events were expected to occur in the subjects. The confidentiality of patients’ identity information and their privacy were guaranteed, and no commercial interest was involved, thus meeting the criteria for exemption from ethical review. The Xinhua Hospital Research Ethics Committee has provided an official statement confirming this exemption (approval number XHEC-D-2024-119). The prospective observational study was approved by the Xinhua Hospital Research Ethics Committee, along with providing and ethical approval for the use of patient information (approval number XHEC-C-2023-013-1).

## Results

### Characteristics of Data Sets

A total of 395,790 (33.58%) EMRs were included in the study after excluding less meaningful records, such as physical examinations, vision tests, and COVID-19 tests. The median age for the training, testing, and validation data sets was 12 (IQR 4-47). Males accounted for 47.03% (182,420/387,876), 47.74% (1889/3957), and 46.75% (1850/3957) of the training, testing, and validation data sets, respectively, while children accounted for 54.85% (212,740/387,876), 54.76% (2167/3757), and 54.97% (2175/3957), respectively (Table 1).

**Figure 2:**
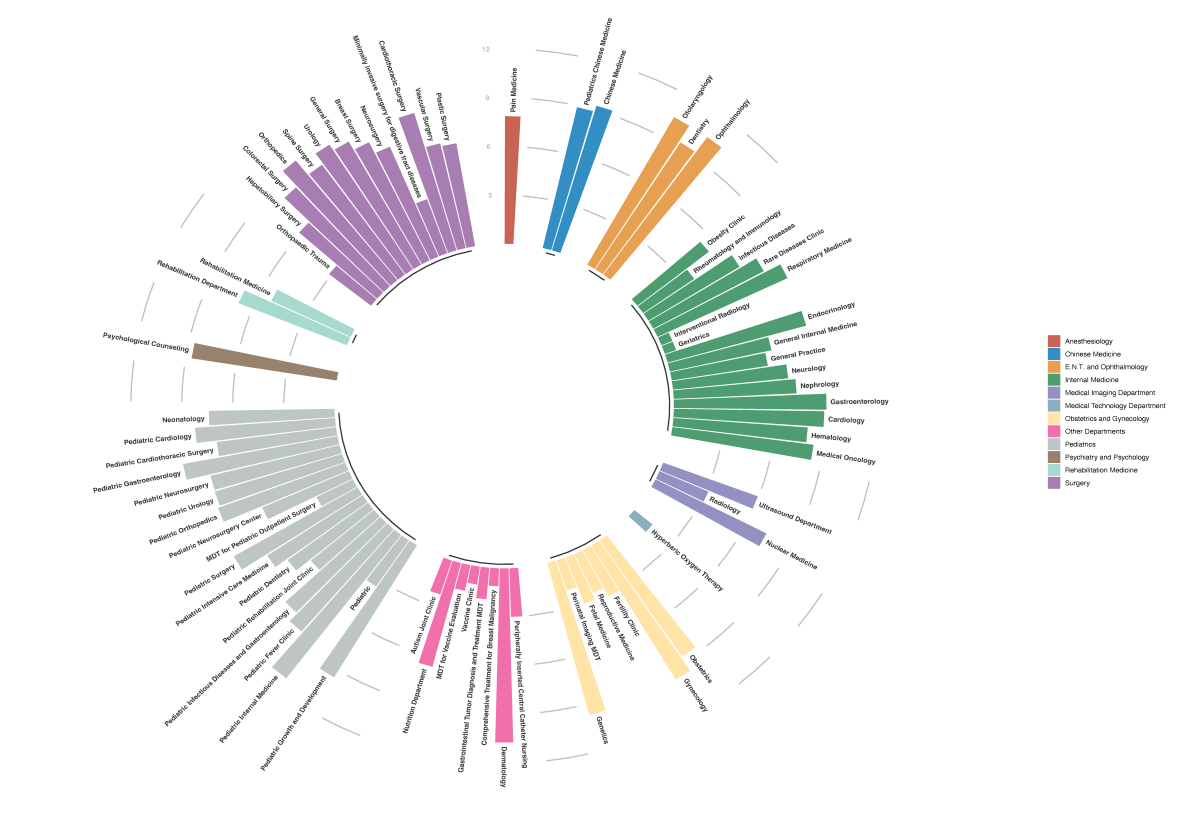
Frequency of departments in EMRs: number of outpatient departments displayed in scale. EMR: electronic medical record; ENT: ear, nose, and throat; MDT: multidisciplinary team.

The EMRs contained 79 subspecialty departments and over 16,000 symptoms (Figure 2). Based on the EMRs, we analyzed the range and frequency of outpatient visits. Overall, the pediatric department has the most outpatient visits (n=117,667, 29.73%) and subspecialty departments (n=19, 24.1%), as shown in Figure 2 and Multimedia Appendix 5. In the obstetrics and gynecology department, genetics, gynecology, and obstetrics were the top 3 recorded departments, with over 15,000 cases in gynecology and obstetrics. In the surgery department, orthopedics, urology, and cardiothoracic surgery were the top 3 recorded departments. In the internal medicine department, gastroenterology, cardiology, and endocrinology were the top 3 recorded departments (Multimedia Appendix 5).

**Table 1:** Clinical characteristics of included data sets.

Characteristics		Training data set (n=387,876)	Testing data set (n=3957)	Validation data set (n=3957)	Implementation data set (n=36,683)
Age (years), median (IQR)		12 (4-47)	12 (5-47)	12 (4-46)	21 (5-35)
**Gender, n (%)**					
	Male	182,420 (47.03)	1889 (47.74)	1850 (46.75)	17,777 (48.46)
	Female	205,456 (52.97)	2068 (52.26)	2107 (53.25)	18,906 (51.54)
**Age group, n (%)**					
	Children	212,740 (54.85)	2167 (54.76)	2175 (54.97)	17,444 (47.55)
	Adults	175,136 (45.15)	1790 (45.24)	1782 (45.03)	19,239 (52.45)

### System Performance Validated Using Testing Data and Validation Data

There are 3 modules in the triage system: patient description extraction, patient description normalization, and department recommendation (Figure 1B). We accessed precision, recall, and the *F*_1_-score based on testing data sets from EMRs and dialogues to evaluate whether the patient description extraction module can accurately extract medical events, such as symptoms and anatomical locations. In the EMR testing data set, the extraction module achieved a precision of 0.8420 for extracting symptoms and 0.7768 for extracting anatomical locations (Multimedia Appendix 6). In the dialogue testing data set, the extraction module achieved a precision of 0.6139 for extracting symptoms and 0.6346 for extracting anatomical locations (Multimedia Appendix 6).

Next, we calculated accuracies of top 1 and 3 recommendations when evaluating system performance. Overall, when training the model on the original data, the model achieved higher accuracies in the original testing and validation data sets. However, when training the model on downsampled data, the results were opposite. In this scenario, the top 3 accuracies were 0.8712 and 0.8641 for the original testing and validation data sets, respectively, and 0.8945 and 0.8941 for the downsampled testing and validation data sets, respectively (Table 2).

**Table 2:** Accuracy (%) of department recommendation in testing and validation data sets.

Data set and groups			Original data-trained model			Downsampled data-trained model	
		First		Top 3	First		Top 3
**Testing**							
	Original	90.79		97.58	72.01		87.12
	Downsampled	89.82		97.06	75.82		89.45
**Validation**							
	Original	90.88		97.4	71.29		86.41
	Downsampled	89.82		97.07	75.71		89.41

### System Performance Validated Using the Cancellation Rate

We implemented the system on the WeChat mini program at the Xinhua Hospital WeChat official account for real-world applications. We observed that the patient cancellation rate reduced from 3.83% (16,037/418,714) in 2021 to 3.53% (15,338/434,200) in 2022 from October 29 to December 5, with *P*<.05. Of note, the age distribution of visits in the application period was similar to that in 2021, with the majority of visits being children (Multimedia Appendix 7). The number of people using the triage system for department recommendations increased from 3590 users in January 2023 to 6953 users in April 2023 according to a review of system usage trends (Multimedia Appendix 8).

### System Performance Validated by Comparing With Triage Nurses

In the prospective observational study, we collected 306 samples to compare the performances of our triage system and triage nurses, of whom 149 (48.7%) were adults and 157 (51.3%) were children. After being evaluated by 3 GPs with over 10 years of experience, it was found that there was a relatively high proportion of patients with pediatric fever (n=69, 22.5%), pediatric patients (n=34, 11.1%), and cardiology patients (n=17, 5.6%). Table 3 summarizes the evaluations. Overall, triage nurses had higher accuracy (98.03%) and precision (92.13%) compared to the triage system (accuracy: 91.53%; precision: 90.49%), while our triage system significantly outperformed in recall@3 (62.30% vs 52.66%, *P*<.001) and time consumption (10.11s vs 14.33 seconds, *P*<.001).

After subgrouping by age, we found that regardless of whether the patients were adults or children, the system still performed better than the triage nurses in the recall@3 rate and average time consumption. In the adult subgroup, the system’s first recommended department precision was 89.19%, which was higher than the precision of triage nurses (87.84%).

**Table 3:** Comparison between triage nurses and the triage system.

Groups and subgroups			Precision (%)		Accuracy (%)		Recall@3^a^ (%)		Time consumption (seconds)
Triage nurses			92.13		98.03^b^		52.66		14.33^b^
Triage system			90.49		91.53		62.30^c^		10.11
**Children (n=157, 51.3%)**									
	Triage nurses	96.18^c^		99.36^d^		51.72		13.79^b^	
	Triage system	91.72		93.63		60.04^e^		10.13	
**Adults (n=149, 48.7%)**									
	Triage nurses	87.84		96.62^f^		53.66		14.91^b^	
	Triage system	89.19		89.30		64.68^g^		10.09	

^a^Recall@3: recall of the top 3 recommended departments.

^b^*P*<.001.

^c^*P*=.031.

^d^*P*=.006.

^e^*P*=.002.

^f^*P*=.013.

^g^*P*=.001.

## Discussion

### Principal Findings

This study developed an AI-powered triage system to generate accurate all-department recommendations for online prehospital registrations. Our main finding was that the triage system was able to make accurate outpatient department recommendations among 79 subspecialty departments in Xinhua Hospital and reduce the time consumption of triage.

In the construction of the triage system, its accuracy for the downsampled testing and validation data sets was 89.45% and 89.41%, respectively, as shown in Table 2, similar to the accuracy in the prospective observational study (91.53%). These results indicate that using downsampling EMRs to simulate patient chief complaints is reliable. During the training process, EMRs were used for accurate labels and substantial chief complaints, while medical dialogue groups were used for the conversion of nonstandard medical terminologies. Dialogue groups were important in extracting medical contents from patient descriptions to reduce the discrepancy between oral expressions and medical terminologies, thereby stabilizing the deep learning model’s output and enhancing its accuracy. Additionally, unlike previous studies that have focused on directly extracting information from EMRs [23-25], we altered the BERT framework in the patient description extraction module to enhance the algorithm framework’s adaptability to Chinese oral expressions [26].

One of the most significant contributions of the triage system is to fulfill all-department recommendations based on patients’ chief complaints, which is suitable for Chinese tertiary hospitals. Most current triage systems focus on emergency department triage, hospitalization predictions, or specialized department triage, such as cardiology and oncology [12,27-30]. In addition, a survey revealed that patients complain about current online registration systems for not knowing which department to choose [31]. Our system can identify many subspecialty departments, while triage nurses or even GPs only recommend main departments. For example, when triage nurses recommend general departments such as pediatrics and cardiology, the proposed system will recommend departments such as pediatric fever and cardiovascular internal medicine. By achieving accurate subspecialty department recommendations, the triage system can improve precision prehospital diagnosis and convenient medical services as patients can precisely choose departments at home [7,11,31].

After implementing the triage system at Xinhua Hospital, we found that the cancellation rate decreased from 3.83% to 3.53% from 2021 to 2022 and the number of triage system users increased from 3590 users in January 2023 to 6953 users in April 2023 (Multimedia Appendix 8). Repeated registrations due to false registrations and long waiting times of the offline outpatient process can lead to patient no-shows, which may cause financial losses and underused medical resources [32,33]. The reduction in the cancellation rate and an increasing number of triage system users may help improve efficiency in the outpatient process and achieve better medical resource allocation, thereby expanding the hospital’s service efficiency. Moreover, such a smartphone-based triage system may further drive the evolution of the “internet plus health care” mode [7], improving the convenience of patient care. With the wider usage of the internet and less cost of distant medical services, the internet hospital, where patients seek consultations over the internet, can further assist medical consultations from diagnosis to treatment recommendation entirely online [2,34].

Our prospective observational study demonstrated that our triage system is comparable to triage nurses, with 91.53% accuracy and 90.49% precision (Table 3). Additionally, our system performs better than triage nurses in the recall@3 rate (62.3%) and average time consumption (10.11 seconds). A higher recall@3 rate indicates that our system recommends more relevant departments. Even though we did not include queue waiting times for comparison, our system’s time consumption was still 3 seconds less than that of triage nurses. This shows the substantial potential of using triage systems to reduce patient waiting times in premedical consultations and improve medical service efficiency. It is possible that the proposed triage system, when integrated with internet hospitals, will fulfill the role of triage nurses in online hospitals and provide convenient medical services.

### Limitations and Future Prospects

Our study has limitations. The first is the limited training data. EMRs were collected from a single center, Xinhua Hospital, in which the departments for pediatric diseases may be more specialized than those in other hospitals. We recognize that department settings vary among hospitals. Meanwhile, with more patient descriptions, our model may perform better for the identification and conversion of symptoms. Second, our triage system is based on the BERT model, which is extensively used for text classification, relation extraction, and entity recognition [26,35,36]. The recent release of Generative Pretrained Transformer 4 (GPT-4) enables inputs as sentences and outputs human-like responses, while BERT can only output word vectors. GPT-4 is better suited for generation tasks, such as medical dialogue generation, generative questions and answers, and automatic medical record filling [37,38]. This may further promote patients’ experiences when they would like to ask more. Third, our system is currently in an early promotional phase, and there is an increasing trend of triage system users, so the influence of the triage system on antual convenient medical service is underestimated. As new systems always need time to become familiar to people, we need to promote the system to more people so that we can vividly see the actual benefit of such a system. Future study is need for long-term investigation. Finally, the comparison between the triage system and triage nurses contains inherent bias. During the experiment, triage nurses directly communicated with patients, while the input of the triage system was generated by rephrasing patients’ chief complaints. In addition, both triage nurses and GPs recommended main departments. During evaluations, the recommended subspecialty department would be considered right if the recommended subspecialty department by the triage system falls under the main departments recommended by GPs. Therefore, recommending departments is more challenging for the triage system. Despite this unfairness, the system is equivalent to triage nurses and capable of triaging in internet hospitals. In the future, more precise labeling should be performed for the comparison to fairly evaluate the performance of the triage system.

### Conclusion

In conclusion, the proposed AI-based triage system can provide more accurate all-department recommendations for a variety of departments, especially subspecialty ones. When implemented in internet hospitals, the system could serve as an effective tool for optimizing the outpatient process with less waiting time. This type of framework holds significant potential for achieving convenient medical service and improving the efficiency of patient care, bridging the gap of the “internet plus health care” mode in China. Although this impact may be seen in a single center now, such successful application may encourage more institutes to collaborate for a comprehensive and universal triage system, fulfilling better usage of medical resources, expanding hospital effectiveness, and providing patients with satisfactory medical services in China.

## Appendix

Multimedia Appendix 1 Detailed construction of the triage system algorithm and application examples.

Multimedia Appendix 2 Number of symptoms in EMRs and patient oral expressions. EMR: electronic medical record.

Multimedia Appendix 3 Symptom extraction example.

Multimedia Appendix 4 Sample size calculation for prospective studies.

Multimedia Appendix 5 Outpatient visits and registrations in main departments and subspecialty departments.

Multimedia Appendix 6 Performance of patient description extraction in EMR testing data set and dialogue testing data set. EMR: electronic medical record.

Multimedia Appendix 7 Age distribution in EMRs and triage system users. EMR: electronic medical record.

Multimedia Appendix 8 Time trend of system users.

Multimedia Appendix 9 Main source code of the triage system.

## Abbreviations

AI: artificial intelligence
BERT: Bidirectional Encoder Representations from Transformers
EMR: electronic medical record
GP: general physician
GPT-4: Generative Pretrained Transformer 4
recall@3: recall of the top 3 recommended departments

## Data Availability

The data sets generated and analyzed during this study are available from the corresponding author upon reasonable request. Codes for the model are available in Multimedia Appendix 9.
