# Supplementary material for: Evolution of the “Internet Plus Health Care” Mode Enabled by Artificial Intelligence: Development and Application of an Outpatient Triage System
Source: J Med Internet Res. 2024 Oct 30;26:e51711. doi: 10.2196/51711 (PMC11561436; doi:10.2196/51711)
Supplement: Multimedia Appendix 1 [file jmir_v26i1e51711_app1.docx]

# Multimedia Appendix 1

# Supplementary Methods

## Introduction to BERT

BERT[1] leverages a bidirectional attention mechanism and a large-scale unsupervised corpus to add contextual and commonsense information to the vector representation of each word in a sentence. Due to its efficient structure and abundant large corpus supply, BERT has achieved state-of-the-art results on various natural language processing tasks such as question answering and classification.

The input to the BERT language model consists of a sequence of tokens S and a mask matrix *MASK*. The token sequence *S*=[*CLS*]+[*x*]^n^+[0]^smax-n-2^, where [*CLS*] is the start symbol used to summarize the sequence overall representation. [*x*]^n^ is the corresponding token sequence after text serialization; [SEP] is the end symbol. [0]^smax-n-2^ is used to fill a text sequence of indeterminate length into a fixed-length sequence. *smax* is the length of the input sequence. The Mask matrix is used to mask the padding portion of the text. If *i , j* < *smax*, *MASK_i , j_*=1, otherwise *MASK_i , j_* =0.

The embedding layer gets a vector representation of all the words in the sequence. It consists of three parts: word embedding *e_word_*, positional embedding *e_pos_* and type embedding *e_type_*. Specifically, word embedding is obtained by the corresponding embedding matrix. Positional embedding is used to capture sequence information, which is ignored in the process of self-attention. Type embedding is used to distinguish between two different sequences of input. Given an input sequence *S*, the initial vector representation of all words in the sequence *H*_0_ is as follows:

$H_{0}=LN\left[ e_{word}\left( S \right)+e_{pos}\left( S \right)+e_{type}\left( S \right) \right]$ （1）

where *LN* stands for layer normalization[2].

*N* multi-head self-attention layers are applied to calculate the context-dependent representations of words (*H_N_*) based on the initial representations (*H*_0_). To solve the problems of gradient vanishing and exploding, ResNet architecture[3] is applied in the layer. In *N* multi-head self-attention layers, every layer can produce the output (*H*_m_) given the previous output of (*m* - 1)-th layer (*H*_m-1_):

$H_{m}^{'}=LN\left[ H_{m-1}+MHSA_{h=12}\left( H_{m-1},MASK \right) \right]$ （2）

$H_{m}=LN\left[ H_{m}^{'}+FFN\left( H_{m}^{'} \right) \right]$ （3）

where $H_{m}^{'}$ indicates intermediate result in the calculation process of *m*-th layer, *MHSA*_h_ and *FFN* represent multi-head self-attention and feed-forward that are defined as follows:

$MHSA_{h}\left( X,MASK \right)=\left[ Att\left( {XW}_{1}^{Q},{XW}_{1}^{K},{XW}_{1}^{V},MASK \right);\cdots;Att\left( {XW}_{h}^{Q},{XW}_{h}^{K},{XW}_{h}^{V},MASK \right) \right]W^{O}$ (4)

$FFN\left( X \right)={GeLU\left( {XW}_{1}+b_{1} \right)W}_{2}+b_{2}$ (5)

where *h* represents the number of self-attention mechanisms in multi-head self-attention layer and *Att* is a single attention mechanism defined as follows:

$Att\left( Q,K,V,MASK \right)=Softmax\left[ \frac{QK^{T}}{\sqrt{d_{k}}}+\left( MASK-1 \right)*\infty\right]V$ (6)

where *Q*, *K* and *V* represent "query'', "key'' and "value'' in the attention calculation process, respectively. Additionally, MASK matrix is used to control the range of attention.

In summary, transformer encoder obtains the corresponding context-dependent representation by inputting the sequence *S* and the MASK matrix:

$H_{N}=transformer-encoder\left( S,MASK \right)$ (7)

Finally, the output of transformer encoder is passed to the corresponding downstream task layer to get the final results.

## Design of Patient Description Extraction Module

The patient description extraction module can be defined as an event extraction task in natural language processing to extract critical medical events from patient dictation.

Specifically, the task can be defined as inputting a piece of patient dictation *S* and outputting the symptom, disease, and examination events. The symptom event contains three slots of "symptom name," "anatomical location," and "existence"; the disease event contains "disease name" and "existence" two slots; the examination event contains "examination name" and "existence" two slots.

Event extraction in the field of natural language processing is usually decomposed into three tasks: entity extraction, entity classification, and relationship classification:

• First, we extract all the values of all the slots in a piece of text through the entity extraction model. This step can be formalized as inputting a piece of text *S* and outputting the values of all slots in it E = {(start pos, end pos, shot name, shot value), ...}. For example, enter the text S = “我的肚子有点痛” (I feel pain in my stomach) and output collection of entity E = {(6, 7, symptom name, 痛 (pain)), (2, 4, anatomical location, 肚子 (stomach)) }. This step extracts the values of all possible slots from the text, which is the basis for all subsequent tasks.

•Secondly, for the three entities of symptom name, disease name, and inspection name in the text, we further judge their existence through the entity classification model. This step can be formalized as inputting a piece of text *S*, and symptom name entities *E_s_*, disease name entities *E_d_*, and examination name entities *E_e_* in it, and outputting the existence of each entity X = {(start pos, end pos, shot name, shot value, existence), ...}. For example, enter the text S = “我肚子不痛” (I don’t feel pain in my stomach) and the entity (6, 7, symptom name, 痛 (pain)), and output the existence condition (6, 7, symptom name, 痛 (pain), nonexistent). This step is used to distinguish positive and negative semantics so that the subsequent department recommendation task can receive more accurate structured patient descriptions.

•Third, we further judge the modification relationship between part and symptom through the relationship classification model for the two entities of symptom name and part name. This step can be formalized as inputting a piece of text *E*, the anatomical location entity *E*_b_ and symptom name entities *E*_s_, and outputting whether there is a modification relationship between the part and the symptom R = {((start pos, end pos, symptom name, shot value), (start pos, end pos, anatomical location, shot value), existence), ...}}. For example, input text S = “我的肚子有点痛” (I feel some pain in my stomach) and output R = {((6, 7, symptom name, 痛 (pain)), (2, 4, anatomical location, 肚子 (stomach)), existent)}. This step is used to determine the association between the symptoms and the parts and to correct the semantics of the symptoms to ensure that the original symptom words, such as “痛” (pain), will not produce semantic deviation.

•Finally, multiple slot values are associated with a core word to form an event. This step can be formalized as inputting the existence of each entity *X* and the relationship *R* and outputting the symptom, disease, and examination events *T*. For example, input X = {(6, 7, symptom name, 痛 (pain), nonexistent)} R = {((6, 7, symptom name, 痛 (pain)), (2, 4, anatomical location, 肚子 (stomach)), existent)} and output T = {symptom: “剧痛” (huge pain), anatomical location: “肚子” (stomach), existence: no}. This step combines and assembles the outputs of the above three models to form an event that subsequent department recommendation model can process in a unified format.

In order to reduce the number of model parameters for the patient description extraction task, we adopted the joint learning method. We used a language model as a shared parameter layer to connect three tasks simultaneously. It can reduce the memory cost during actual use and improve the model's performance by using the interaction information between tasks. The architecture of the extraction model is demonstrated in the Figure S1. It contains layers of language model encoder, entity extraction downstream task, entity classification downstream task and relation classification downstream task.

The language model encoder layer directly uses the encoder layer of the BERT language model, so that the model can directly use a large amount of knowledge learned by the language model in the pre-training process. It contains an embedded layer and *N* multi-head self-attention layers. For the language model, we only need to obtain the output of the last Nth layer *H_N_* as the task representation *H*_task_ and pass it to the specific task layer.

$H_{task}=H_{N}=transformer-encoder\left( S,MASK \right)$ (8)

Subsequently, we will introduce the construction downstream task layer of entity extraction, entity classification and relation classification in blocks.

*1) The Construction of Entity Extraction Downstream Task Layer:* In the entity extraction task, the downstream task layer needs to convert the representation vector of the task representation *H_task_* into the probability distribution of whether each span (*i, j*) is a specific type of entity *P_etype,i,j_*, where the span is defined as a tuple including the start position and the end position. We use the GlobalPointer[4] structure to achieve this goal (Figure S1). Compared with the traditional CRF structure[5], this structure can better represent the nested relationship between entities, which is common in the medical field. For example, in the symptom entity “下肢疼痛” (lower limbs pain), there is a anatomical location entity “下肢” (lower limbs).

Specifically, GlobalPointer imitates the calculation method of multi-head self-attention and uses the attention head to calculate the probability distribution of each entity span. The input task represents *H_task_*: First, using two fully connected layers, map to *Q_etype_* and *K_etype_*.

$Q_{etype}=H_{task}\times W_{etype}^{Q}+b_{etype}^{Q}$ (9)

$K_{etype}=H_{task}\times W_{etype}^{K}+b_{etype}^{K}$ (10)

where $W_{etype}^{Q},W_{etype}^{K}\in\mathbb{R}_{h\times d},b_{etype}^{Q},b_{etype}^{K}\in\mathbb{R}_{d}$, *h* represents the original word vector dimension of *H_task_*, and *d* represents the transformed dimension of the fully connected layer.

Second, superimpose the rotary position embedding (RoPE)[6] to introduce position information.

$Q_{etype}^{'}=RoPE\left( Q_{etype} \right)$ (11)

$K_{etype}^{'}=RoPE\left( K_{etype} \right)$ (12)

Then do the dot product of $Q_{etype}^{'}$ and $K_{etype}^{'}$ to get a scoring matrix *A_etype_* for each span.

$A_{etype}=\frac{Q_{etype}^{'}\times{K_{etype}^{'}}^{T}}{\sqrt{d_{k}}}$ (13)

After a Sigmod transformation, the corresponding probability distribution can be obtained.

$P_{etype}=\frac{1}{1+e^{-A_{etype}}}$ (14)

Due to entities' highly unbalanced probability distribution, most tuples in the text are not entities. This leads to negative examples being much more significant than positive examples. Therefore, we automatically use Circle Loss[7] to balance the weight between positive and negative sample.

$L_{etype}=Log\left[ 1+\sum_{i,j\in entities} -A_{etype,i,j} \right]+Log\left[ 1+\sum_{i,j\notin entities} A_{etype,i,j} \right]$ (15)

*The Construction of Entity Classification Downstream Task Layer*: In the entity classification task, the downstream task layer also needs to convert the representation vector of the task representation *H_task_* into the probability distribution of whether the span of each entity exists. We also use the GlobalPointer structure to achieve this goal, and its calculation process is same as Eq. (9)-(14). Finally, we get the scoring matrix *A_existence_* of entity existence and the corresponding probability distribution *P_existence_*. Its loss function is also implemented by a similar Circle Loss, as follows:

$L_{existence}=Log\left[ 1+\sum_{i,j\in existing entities} -A_{existence,i,j} \right]+Log\left[ 1+\sum_{i,j\in non-existent entities} A_{existence,i,j} \right]$ (16)

The Construction of Relation Classification Downstream Task Layer: For the relation classification task, it is necessary to judge whether there is a relationship between two spans (symptom start pos, symptom end pos, symptom name, symptom shot value) and (anatomical location start pos, anatomical location end pos, anatomical location, anatomical location shot value). We can transform it into a probability distribution that judges whether there is a relationship between two spans (symptom start pos, anatomical location start pos) and (symptom end pos, anatomical location end pos). Therefore, GlobalPointer can also be used for modeling to obtain the score *A*_relation_ and the probability distribution *P*_relation_. Its loss function is also implemented by a similar Circle Loss, as follows:

$L_{relation}=Log\left[ 1+\sum_{si,bi\in exist relation} -A_{relation,si,bi} \right]+Log\left[ 1+\sum_{sj,bj\in exist relation} -A_{relation,sj,bj} \right]+Log\left[ 1+\sum_{si,bi\in non relation} A_{relation,si,bi} \right]+Log\left[ 1+\sum_{sj,bj\in non relation} A_{relation,sj,bj} \right]$ (17)

The loss function of the joint model (i.e., *L_all_*) will be obtained as follows:

$L_{all}=L_{etype}+L_{existence}+L_{relation}$ (18)

where *L_etype_*, *L_existence_* and *L_relation_* are defined in Eq. (15), (16) and (17), respectively.

In summary, this structure has two advantages:

It obtains the representation vector of the task through the strong feature extraction ability of BERT. Compared with the complex representation conversion layer, the structure is easier to optimize.

It does not significantly adjust the structure of the BERT language model, so the structure can directly use the prior knowledge contained in the parameters of pre-trained language model.

## Design of Description Normalization Module

The patient description standardization module aims that the entity name in medical events will be mapped to a standard term corresponding to the concept it refers to. The entity name includes three types: symptom name, disease name and anatomical location. Specifically, the task can be defined as inputting a specific entity text *E*, and the standard terminology collection *N*, and outputting standard words *S* in *N*. For example, input *E* = “经腹右肾上腺嗜铬细胞瘤切除术” (Right adrenal pheochromocytoma resection), and *N*=*ICD*10, output standard words *S* = “肾上腺病损切除术” (Adrenal lesion resection).

Implementing a patient description standardization module requires two steps. We first build a standard terminology collection and then build the corresponding standardized model.

*1) The Construction of Standard Terminology Collection:* For the standard terminology collection of disease names, we directly adopted the tenth edition of the International Classification of Diseases, Injuries and Causes of Death (ICD-10). ICD-10 is classified according to the site of disease occurrence. Concepts between different levels are connected using the Is-A (belongs to) relationship. In order to simplify the task, the Is-A relationship in the terminology system is ignored here, and the terminology system is only regarded as a collection of standard terminology words. It is only necessary to map the entity words to the standard terminology collection in the set without considering the tree structure formed by the standard terminology collection. Statistics are as Table S1.

## Table S1. Statistics of standard terminology collection for ICD-10

| **Entity Type** | **Terminology Number** |
| --- | --- |
| **Disease Name** | 37 344 |
| **Total** | 37 344 |

There is no unified standard in China for the standard terminology collection of symptom names and anatomical locations. Therefore, we use the entity extraction algorithm in Section S1b to extract entities from the collected medical record data. After statistics, clustering, and labelling, new standard terminology collection for symptom name and anatomical location are formed. Statistics are as Table S2.

## Table S2. Statistics of standard terminology collection for symptom name and anatomical location

| **Entity Type** | **Terminology Number** | **Synonyms Number** |
| --- | --- | --- |
| **Anatomical location** | 731 | 8717 |
| **Symptom Name** | 9183 | 13 408 |
| **Total** | 9914 | 22 125 |

*2) The Construction of Standardized Model:* We adopt a contrastive learning method to model the standardized model. The goal of contrastive learning is to learn an encoder that encodes standard entity words and non-standard entity words into vectors in a high-dimensional space. If entity text *E* and outputting standard word *S* are synonymous, the distance between the vectors is relatively close; otherwise, the distance between the vectors is farther.

Specifically, we use BERT-whitening as our encoder and cos distance as the measurement function of the distance between vectors. Figure S2 shows the model's architecture. It consists of two parts, the language model encoder layer and the whitening layer.

The language model encoder layer, similar to the Section S1b, directly uses the encoder layer of the BERT language model. This enables the model to directly use a large amount of knowledge learned by the language model during the pre-training process. For contrastive learning, we obtain the output *H*_0_ of the first layer and the output of the last Nth layer *H_N_*, then pass it into the MaxPooling layer as the output of the encoder *H_W_*.

$H_{W}=maxpooling\left( concat\left( H_{0},H_{N} \right) \right)$ (19)

The whitening layer transforms the mean value of all encoder outputs to 0 and transforms the covariance matrix into an identity matrix, including standard and non-standard words. It is equivalent to the whitening operation in data mining.

Specifically, we first calculate the mean of the encoder outputs of all words $\overline{H_{W}}$.

$\overline{H_{W}}=\frac{\sum_{W\in words} H_{W}}{len\left( words \right)}$ (20)

Next, we use SVD to find the corresponding transformation matrix.

$\Sigma=\frac{\sum_{W\in words} \left( H_{W}-\overline{H_{W}} \right)^{T}\left( H_{W}-\overline{H_{W}} \right)}{len\left( words \right)}$ (21)

$\Sigma=U\Lambda U^{\top}$ (22)

$W_{white}=U\sqrt{\Lambda^{-1}}$ (23)

Finally, we get the final output $H_{W}^{'}$.

$H_{W}^{'}=\left( H_{W}-\overline{H_{W}} \right)\times W_{white}$ (24)

We then compute the cos similarity between the representation of the original word and the representations of all terms in the standard terminology collection *N* and rank them. The term with the highest similarity can be regarded as the standard term of the original word.

## Design of Department Recommendation Module

The department recommendation module aims to classify the medical events processed by the above two modules into departments. This module is responsible for the final output and is the core of the whole process. Specifically, this task can be defined as inputting the standardized medical event *T*, the patient's age *Age*, the patient's gender *Gender*, and outputting the corresponding recommended medical department *D*. For example, input T = {symptom: “疼痛” (pain), anatomical location: “腹部” (abdomen), existence: yes}, {symptom: “月经失调” (menstrual dysregulation), anatomical location: none, existence: yes}, Age = “28”, Gender = “女” (female), output department: *D* = “妇科” (Gynecology).

Implementing a department recommendation module requires two steps. First, we converted medical events, age and gender into standard medical chief complaint using a template. Second, we build a chief complaint classification model, input the chief complaint text, and output the corresponding department.

*1) Patient Chief Complaint Generation:* Patient Chief Complaint Generation essentially converts medical events *T*, the patient's age *Age* and the patient's gender *Gender* into standard medical chief complaint texts *TS*. For example, when we input T = {symptom: “疼痛” (pain), anatomical location: “腹部” (abdomen), existence: no}, {symptom: “月经失调” (menstrual dysregulation), anatomical location: none, existence: no}, Age = “28岁”, Gender = “女” (female), the converted chief complaint text TS = “患者，女，腹部无疼痛，月经失调。” (Patient: female, no abdominal pain, menstrual dysregulations).

The complete generation process of the chief complaint text is shown in Algorithm S1.

| **Algorithm S1** Generate Main Complaint Text |
| --- |
| **Require**: medical events *T*, patient’s age *Age* and patient’s gender *Gender*  **Ensure**: chief complaint text *TS*  *TS* ← “”  **if** *Age* <18 **then**  *TS* ← *TS* + “患儿” (child patient)  **else if** Age > 60 **then**  *TS* ← *TS* + “老年患者” (elderly patient)  **else**  *TS* ← *TS* + “患者” (patient)  **end if**  *TS* ← *TS* + “,”  **if** *Gender* = “男” (male) **then**  *TS* ← *TS* + “男” (male)  **else**  *TS* ← *TS* + “女” (female)  **end if**  *TS* ← *TS* + “,”  **for** *T_sym_* in *T* **do**  *TS* ← *TS* + $T_{sym}^{anatomical location}$  **if** $T_{sym}^{existence}$= “no” **then**  *TS* ← *TS* + “无” (no)  **end if**  *TS* ← *TS* + $T_{sym}^{name}$+ “,”  **end for**  **for** *T_dis_* in *T* **do**  **if** $T_{dis}^{existence}$ = “no” **then**  *TS* ← *TS* + “无”  **end if**  *TS* ← *TS* + $T_{dis}^{name}$+ “,”  **end for**  *TS*[-1] ← “。” |

Algorithm S1 converts non-standard patient oral information into standard chief complaint text through template generation. This way, we can convert the department classification task into a text classification task and use the language model to solve it.

**2)The Construction of Chief Complaint Classification Model:** We adopted the language model superposition MLP to construct the chief complaint classification model. Figure S2 shows the model's architecture. It consists of two parts, the language model encoder layer and the MLP layer. The language model encoder also uses the structures defined in Section S1c. We also need to obtain the output of the last Nth layer *H_N_* as the task representation *H_c_* and pass it to the MLP layer.

$H_{c}=H_{N}=transformer-encoder\left( S,MASK \right)$ (25)

The MLP layer is essentially a nonlinear combination of two fully connected layers. It converts the task representation *H_c_* output by the language model into the probability distribution *P_c_* of the department.

$H_{c}^{'}=Gelu\left( H_{c}\times W_{c}^{1}+b_{c}^{1} \right)$ (26)

$P_{c}=Softmax\left( H_{c}^{'}\times W_{c}^{2}+b_{c}^{2} \right)$ (27)

where $W_{c}^{1}\in\mathbb{R}_{h\times h},b_{c}^{1}\in\mathbb{R}_{h},W_{c}^{2}\in\mathbb{C}_{h\times h},b_{c}^{2}\in\mathbb{R}_{C}$, *h* represents the original word vector dimension of *H_task_*, and *C* represents number of departments. The Gelu[8] function is a non-linear activation function between two fully connected layers. The Softmax function converts the output of the second fully connected layer into a probability distribution.

Because the probability distribution of departments is also unbalanced. Therefore, we use Focal Loss[9] to balance the weights among samples from different departments.

$L_{c}=-\left( 1-P_{c} \right)^{g}Log\left( P_{c} \right)$ (28)

where *g* = 2 refers to the setting of the Focal Loss paper.

## Evaluation of Patient Description Extraction

We evaluated performance of patient description extraction in testing dataset of EHRs and dialogue dataset. The performance was evaluated through calculating precision, recall and F1-score regarding diagnosis name, symptom name, anatomical locations, and symptom and anatomical location associations.

## Figure S1. Entity Extraction of the Triage system


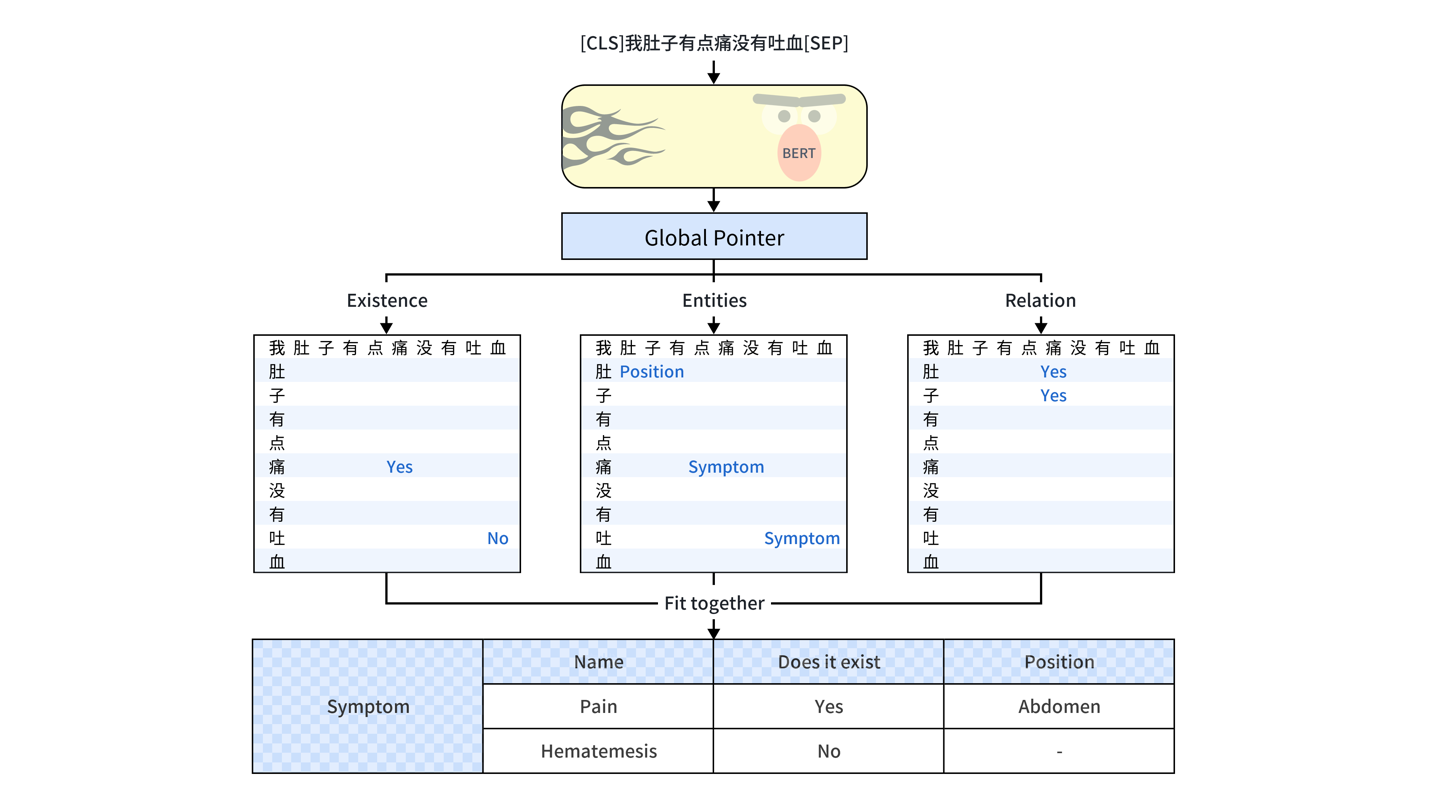


*我肚子有点痛没有吐血(I have a stomachache and no vomiting blood)

## Figure S2. Algorithm Models of the Triage system


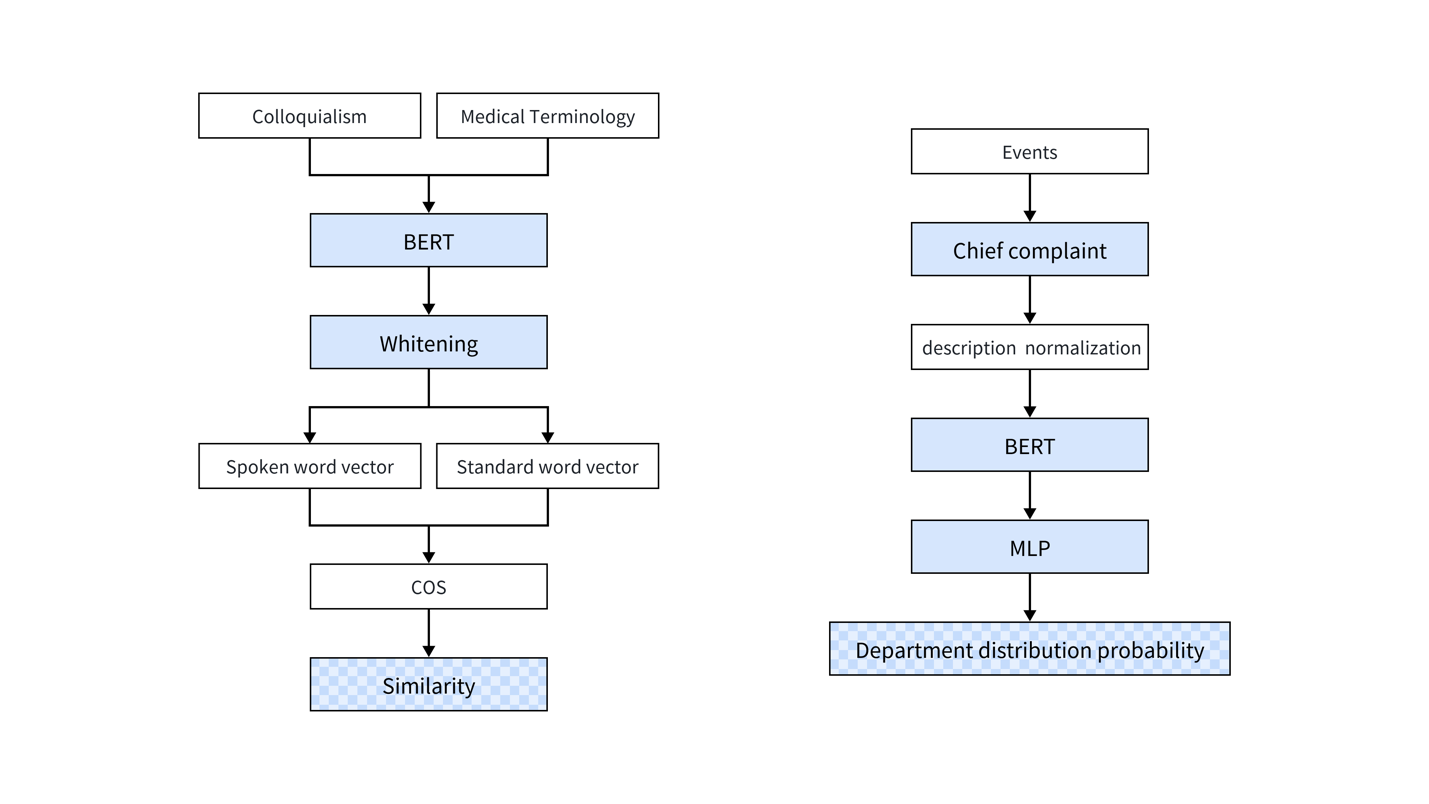


## References

1. Devlin J, Chang M-W, Lee K, Toutanova K. BERT: Pre-training of Deep Bidirectional Transformers for Language Understanding. arXiv; 2019. doi: 10.48550/arXiv.1810.04805

2. Ba JL, Kiros JR, Hinton GE. Layer Normalization. arXiv; 2016. doi: 10.48550/arXiv.1607.06450

3. He K, Zhang X, Ren S, Sun J. Deep Residual Learning for Image Recognition. 2016. p. 770–778. Available from: https://openaccess.thecvf.com/content_cvpr_2016/html/He_Deep_Residual_Learning_CVPR_2016_paper.html [accessed Jul 13, 2023]

4. Su J, Murtadha A, Pan S, Hou J, Sun J, Huang W, Wen B, Liu Y. Global Pointer: Novel Efficient Span-based Approach for Named Entity Recognition. arXiv; 2022. doi: 10.48550/arXiv.2208.03054

5. Huang Z, Xu W, Yu K. Bidirectional LSTM-CRF Models for Sequence Tagging. arXiv; 2015. doi: 10.48550/arXiv.1508.01991

6. Su J, Lu Y, Pan S, Murtadha A, Wen B, Liu Y. RoFormer: Enhanced Transformer with Rotary Position Embedding. arXiv; 2022. doi: 10.48550/arXiv.2104.09864

7. Sun Y, Cheng C, Zhang Y, Zhang C, Zheng L, Wang Z, Wei Y. Circle Loss: A Unified Perspective of Pair Similarity Optimization. 2020. p. 6398–6407. Available from: https://openaccess.thecvf.com/content_CVPR_2020/html/Sun_Circle_Loss_A_Unified_Perspective_of_Pair_Similarity_Optimization_CVPR_2020_paper.html [accessed Jul 13, 2023]

8. Hendrycks D, Gimpel K. Gaussian Error Linear Units (GELUs). arXiv; 2023. doi: 10.48550/arXiv.1606.08415

9. Lin T-Y, Goyal P, Girshick R, He K, Dollar P. Focal Loss for Dense Object Detection. 2017. p. 2980–2988. Available from: https://openaccess.thecvf.com/content_iccv_2017/html/Lin_Focal_Loss_for_ICCV_2017_paper.html [accessed Jul 13, 2023]

**Example of Mobile Implementation:**

The triage system was implemented in Xihua Hospital Wechat Official Account:


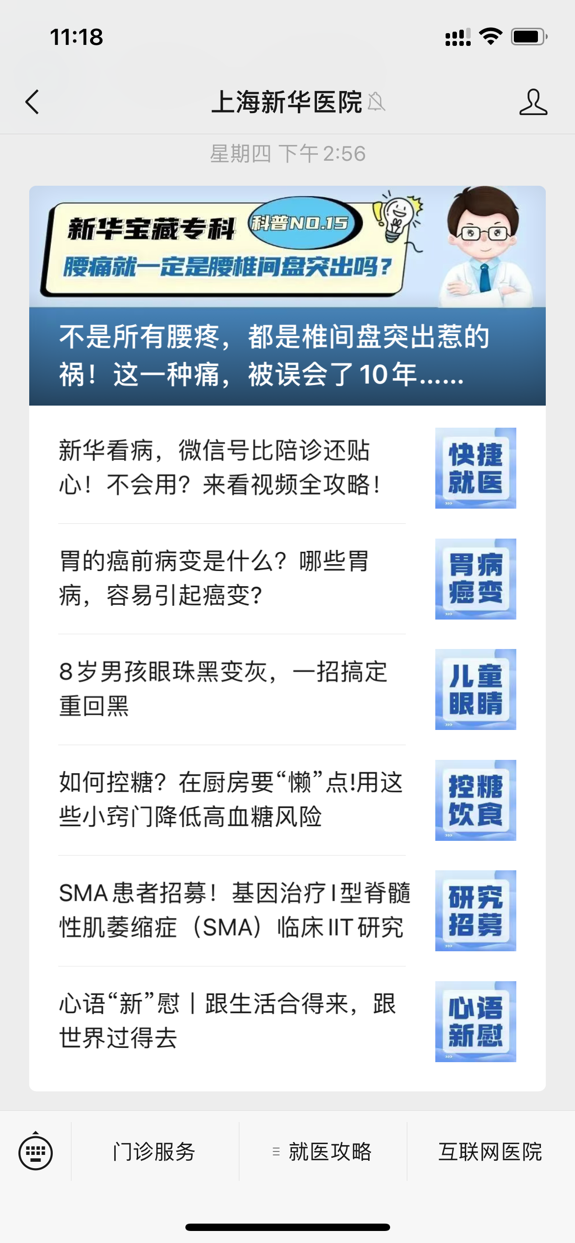

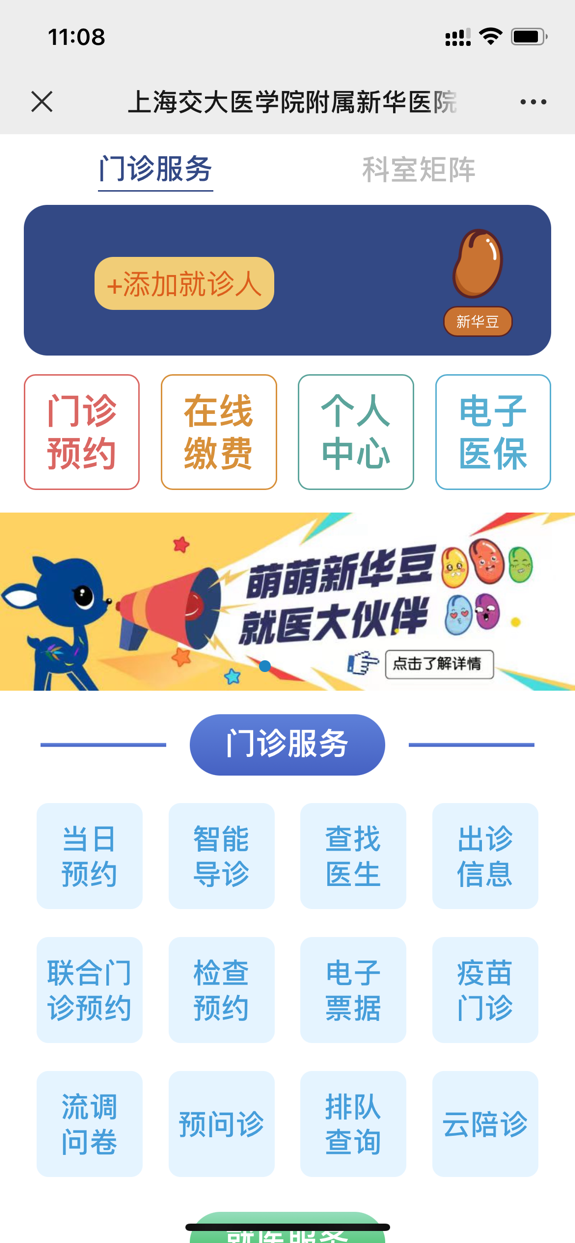


(3)

(2)

(1)

(1). Xinhua Hospital Wechat Official Account

(2). Outpatient Service

(3). Intelligent Triage


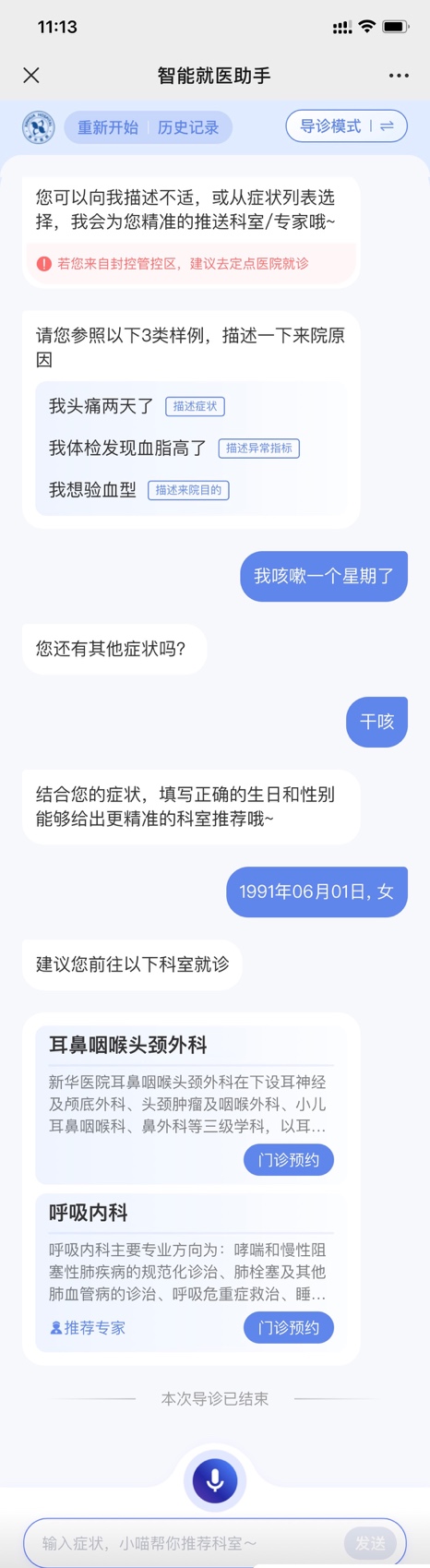

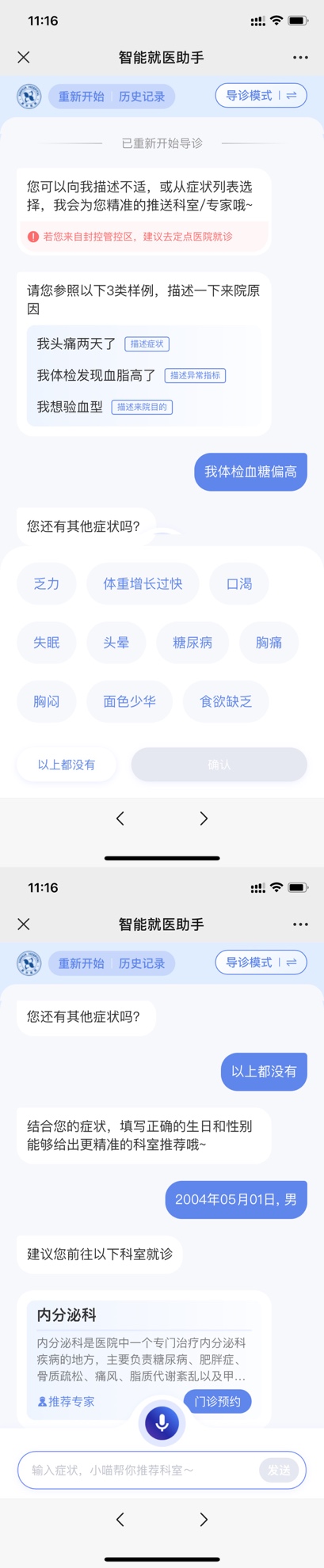


**Chat 1 Translation:**

System: You can describe your discomfort to me, or choose from the list of symptoms, and I will accurately recommend departments/experts for you.

Please refer to the following 3 types of examples to describe the reason for coming to the hospital.

I’ve had a headache for two days (describe the symptoms)

My physical examination revealed that my blood lipids were high (describe abnormal examination results)

I want to have my blood type tested (describe the purpose of coming to the hospital)

Patient: I’ve been coughing for a week

System: Do you have any other symptoms?

Patient: Dry cough

System: Based on your symptoms, filling in the correct date of birth and gender can provide a more accurate department recommendation.

Patient: June 1st, 1991, Female

System: It is recommended that you go to the following department for treatment: Otolaryngology-Head and Neck Surgery, Respiratory medicine

**Chat 2 Translation:**

System: You can describe your discomfort to me, or choose from the list of symptoms, and I will accurately recommend departments/experts for you.

Please refer to the following 3 types of examples to describe the reason for coming to the hospital.

I’ve had a headache for two days (describe the symptoms)

My physical examination revealed that my blood lipids were high (describe abnormal examination results)

I want to have my blood type tested (describe the purpose of coming to the hospital)

Patient: I found my blood sugar is high after physical examination.

System: Do you have any other symptoms? (Choosing from: Tiredness; Gaining weight quickly; Thirsty; Insomnia; Dizziness; Diabetes; Chest pain; Chest distress; Pale; Lack of appetite; None of the above)

Patient: None of the above

System: Based on your symptoms, filling in the correct date of birth and gender can provide a more accurate department recommendation.

Patient: May 1st, 2004, Male

System: It is recommended that you go to the following department for treatment: Endocrinology
