# Supplementary material for: Evolution of the “Internet Plus Health Care” Mode Enabled by Artificial Intelligence: Development and Application of an Outpatient Triage System
Source: J Med Internet Res. 2024 Oct 30;26:e51711. doi: 10.2196/51711 (PMC11561436; doi:10.2196/51711)
Supplement: Multimedia Appendix 2 [file jmir_v26i1e51711_app2.docx]

## Multimedia Appendix 2

## Table S3. Number of symptoms in EMRs.

| **Number of Symptoms** | **Count (%)** |
| --- | --- |
| 1 | 111 029 (15.01) |
| 2 | 125 139 (16.94) |
| 3 | 81 336 (11.01) |
| 4 | 68 751 (9.30) |
| 5 | 70 010 (9.48) |
| 6 | 72 082 (9.76) |
| 7 | 43 592 (5.90) |
| 8 | 30 246 (4.09) |
| 9 | 27 684 (3.75) |
| 10 | 35 239 (4.77) |
| 11 | 23 203 (3.14) |
| 12 | 18 862 (2.55) |
| 13 | 9271 (1.25) |
| 14 | 6832 (0.92) |
| 15 | 3910 (0.53) |
| 16 | 3061 (0.41) |
| 17 | 6233 (0.84) |
| 18 | 1206 (0.16) |
| 19 | 1203 (0.16) |
| 20 | 685 (0.09) |

## Table S4. Number of symptoms in patient expressions.

| **Number of Symptoms** | **Count (%)** |
| --- | --- |
| 0 | 13 478 (36.74) |
| 1 | 18 010 (49.10) |
| 2 | 3 724 (10.15) |
| 3 | 990 (2.70) |
| 4 | 300 (0.82) |
| 5 | 111 (0.30) |
| 6 | 29 (0.08) |
| 7 | 22 (0.06) |
| 8 | 6 (0.02) |
| 9 | 8 (0.02) |
| 10 | 0 (0.00) |
| 11 | 4 (0.01) |
| 12 | 0 (0.00) |
| 13 | 1 (0.00) |
| 14 | 0 (0.00) |
