# Supplementary material for: Evolution of the “Internet Plus Health Care” Mode Enabled by Artificial Intelligence: Development and Application of an Outpatient Triage System
Source: J Med Internet Res. 2024 Oct 30;26:e51711. doi: 10.2196/51711 (PMC11561436; doi:10.2196/51711)
Supplement: Multimedia Appendix 3 [file jmir_v26i1e51711_app3.docx]

**Training Input Snippets**

[CLS] 女性，家住陈栅子乡化育沟村。主因右股内侧红肿疼痛3天于2016-10-15；19：09收入院。[SEP]

输出：

[

[

"部位",

16,

20,

"右股内侧",

"股内侧"

],

[

"症状名称",

20,

22,

"红肿",

"红肿"

],

[

"症状名称",

22,

[

"症状名称",

20,

22,

"红肿",

"红肿"

],

[

"症状名称",

22,

24,

"疼痛",

"疼痛"

]

]

**Downsampling Example:**

Original EMR:

'患者，男，胸部胸闷，咳嗽，无贫血，无肌性肌无力，患有多尿、生殖细胞瘤、骨髓增生、肿瘤、畸胎瘤。已行胸部CT、胸片、心脏超声、胃镜、血常规、血生化、凝血常规检查。曾行鞍区肿瘤切除术。曾使用健儿清解液、头孢克肟药物。存在内胚窦瘤史。'

Downsampling:

'患者，男，胸部胸闷，咳嗽，无贫血，无肌性肌无力，患有生殖细胞瘤、肿瘤。曾使用健儿清解液、头孢克肟药物。'

**The translation of the above is provided for the reader's convenience.**

Training Input Snippets

[CLS] female, living in Huayugou Village, Chenzhazi Township. Suffering redness, swelling and pain on the medial right thigh for 3 days. Admission to the hospital on 2016-10-15; 19:09. [SEP]

Output of Entity extraction：

[

[

"location",

16,

20,

"Right medial femur",

"Medial femur "

],

[

"Symptom",

20,

22,

"Redness and swelling",

"Redness and swelling"

],

[

"Symptom",

22,

[

"Symptom",

20,

22,

"Redness and swelling",

"Redness and swelling"

],

[

"Symptom",

22,

24,

"Pain",

"Pain"

]

]

The output will be used for department recommendation.

**Downsampling Example:**

Original EMR:

'Patient, male, chest tightness, cough, no anemia, no muscular muscle weakness, polyuria, germ cell tumor, myelohyperplasia, tumor, teratoma. Chest CT, chest X-ray, cardiac ultrasound, gastroscopy, blood routine, blood biochemistry, and coagulation routine examinations have been performed. He has undergone sellar tumor resection. He has used Jian'er antidote and cefixime. Presence of a history of endodermal sinus tumor. '

Downsampling:

' patient, male, chest tightness, cough, no anemia, no muscle weakness, germ cell tumor, tumor. He has used Jian'er antidote and cefixime.'
