# Supplementary material for: Evolution of the “Internet Plus Health Care” Mode Enabled by Artificial Intelligence: Development and Application of an Outpatient Triage System
Source: J Med Internet Res. 2024 Oct 30;26:e51711. doi: 10.2196/51711 (PMC11561436; doi:10.2196/51711)
Supplement: Multimedia Appendix 4 [file jmir_v26i1e51711_app4.docx]

The study aims to conduct a self-controlled comparison, evaluating the accuracy of department recommendations for cases with the same symptoms through nurses’ consultation versus triage system guidance. The expected outcomes were summarized in the 2×2 table below.

|  |  | Nurses | |  |
| --- | --- | --- | --- | --- |
|  |  | True | False | Summary |
| System | True | P11 | P10 | $P_{t}$ |
|  | False | P01 | P00 | ${1-P}_{t}$ |
|  | Summary | $P_{s}$ | ${1-P}_{s}$ | 1 |

According to the formula for calculating sample size for the tests for two paired proportions (the McNemar test),

$$N=\frac{\left\{ Z_{1-\frac{\alpha}{2}}\left( OR+1 \right)+Z_{1-\beta}\sqrt{{(OR+1)}^{2}-{(OR-1)}^{2}PD} \right\}^{2}}{{(OR-1)}^{2}PD}$$

$$\rho=\frac{P11-P_{s}P_{t}}{\sqrt{P_{s}P_{t}(1-P_{s})(1-P_{t})}}$$

Where $OR=\frac{P10}{P01}, PD=P10+P01$. Based on prior knowledge, the accuracy of recommendations from triage nurse $P_{s}$ is 90%, and the system's recommendation accuracy $P_{t}$ is 97%. Type I error is set at 0.05, statistical power at 0.9, $\rho$ set to 0.5, resulting in a calculated sample size of 152. Considering the large number of pediatric patients at Xinhua Hospital and the high proportion of pediatric consultations through the triage system (pediatric consultations account for 50.85%), the final sample size is set at 300, with 150 samples allocated to pediatric cases and 150 to adult cases.

Note: *ρ* is the within-subject correlation coefficient. Using this relationship, values of *ρ* can be entered and transformed to the corresponding value of P11 using the equation. In our study, ρ set to 0.5. The values of OR and PD can be calculated as 51 and 0.728.
