# Supplementary material for: Evolution of the “Internet Plus Health Care” Mode Enabled by Artificial Intelligence: Development and Application of an Outpatient Triage System
Source: J Med Internet Res. 2024 Oct 30;26:e51711. doi: 10.2196/51711 (PMC11561436; doi:10.2196/51711)
Supplement: Multimedia Appendix 5 [file jmir_v26i1e51711_app5.docx]

# Multimedia Appendix 5

## **Figure S3.** Number of registrations in main departments and subspecialty departments.


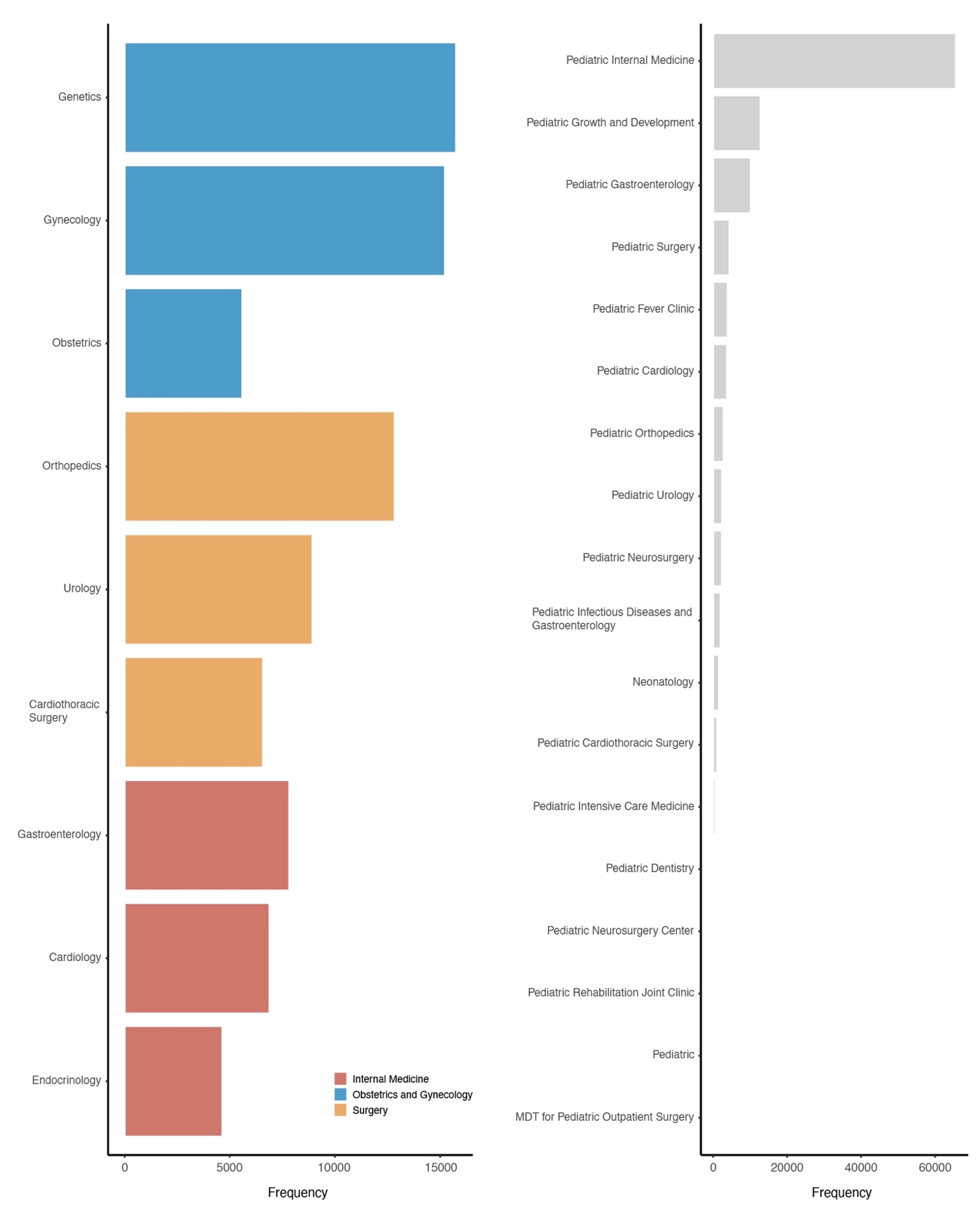


The bar plots summarize registrations of departments recorded in Electronic Medical Records. The left plot shows top 3 recorded departments in Internal Medicine Department, Surgery Department, and Obstetrics and Gynecology Department. The right plot shows recorded departments in Pediatric Department.

## **Table S5.** Number of outpatient visits in main departments.

| **Department** | **Number of Visits (%)** | **Subspecialty** |
| --- | --- | --- |
| **Internal Medicine** | 37 112 (9.38) | 16 |
| **Surgery** | 49 529 (12.51) | 13 |
| **Obstetrics and Gynecology** | 37 345 (9.44) | 7 |
| **Pediatrics** | 117 667 (29.73) | 19 |
| **Other Departments** | 154 137 (38.94) | 24 |
