# Supplementary material for: Evolution of the “Internet Plus Health Care” Mode Enabled by Artificial Intelligence: Development and Application of an Outpatient Triage System
Source: J Med Internet Res. 2024 Oct 30;26:e51711. doi: 10.2196/51711 (PMC11561436; doi:10.2196/51711)
Supplement: Multimedia Appendix 6 [file jmir_v26i1e51711_app6.docx]

# **Multimedia Appendix 6**

## Table S6. Performance of patient description extraction in EMRs testing data set.

|  | **Precision** | **Recall** | **F1-score** |
| --- | --- | --- | --- |
| **Diagnosis name** | 0.8206 | 0.7790 | 0.7992 |
| **Symptom name** | 0.8420 | 0.8245 | 0.8332 |
| **Anatomical locations** | 0.7768 | 0.7471 | 0.7617 |
| **Symptom and anatomical location associations** | 0.7024 | 0.6230 | 0.6603 |

## Table S7. Performance of patient description extraction in dialogue testing data set.

|  | **Precision** | **Recall** | **F1-score** |
| --- | --- | --- | --- |
| **Diagnosis name** | 0.8123 | 0.8351 | 0.8235 |
| **Symptom name** | 0.6139 | 0.5770 | 0.5949 |
| **Anatomical locations** | 0.6346 | 0.7163 | 0.6730 |
| **Symptom and anatomical location associations** | 0.3053 | 0.2685 | 0.2857 |
