# Supplementary material for: Evolution of the “Internet Plus Health Care” Mode Enabled by Artificial Intelligence: Development and Application of an Outpatient Triage System
Source: J Med Internet Res. 2024 Oct 30;26:e51711. doi: 10.2196/51711 (PMC11561436; doi:10.2196/51711)
Supplement: Multimedia Appendix 7 [file jmir_v26i1e51711_app7.docx]

## Multimedia Appendix 7

## Figure S4. Age distribution in EMRs.


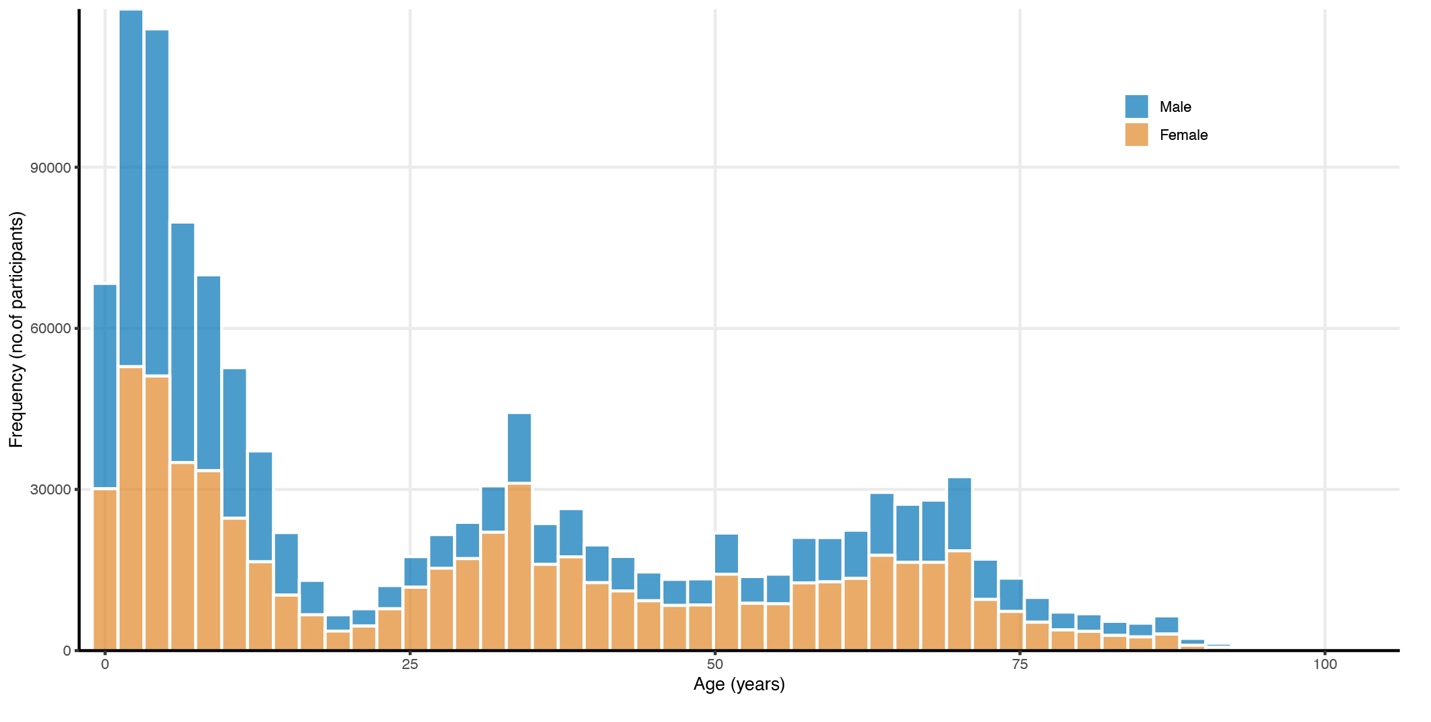


EMRs is abbreviation of Electronic Medical Records. The bar plot shows number of participants of different genders in EMRs.

## Figure S5. Age distribution of triage system users.


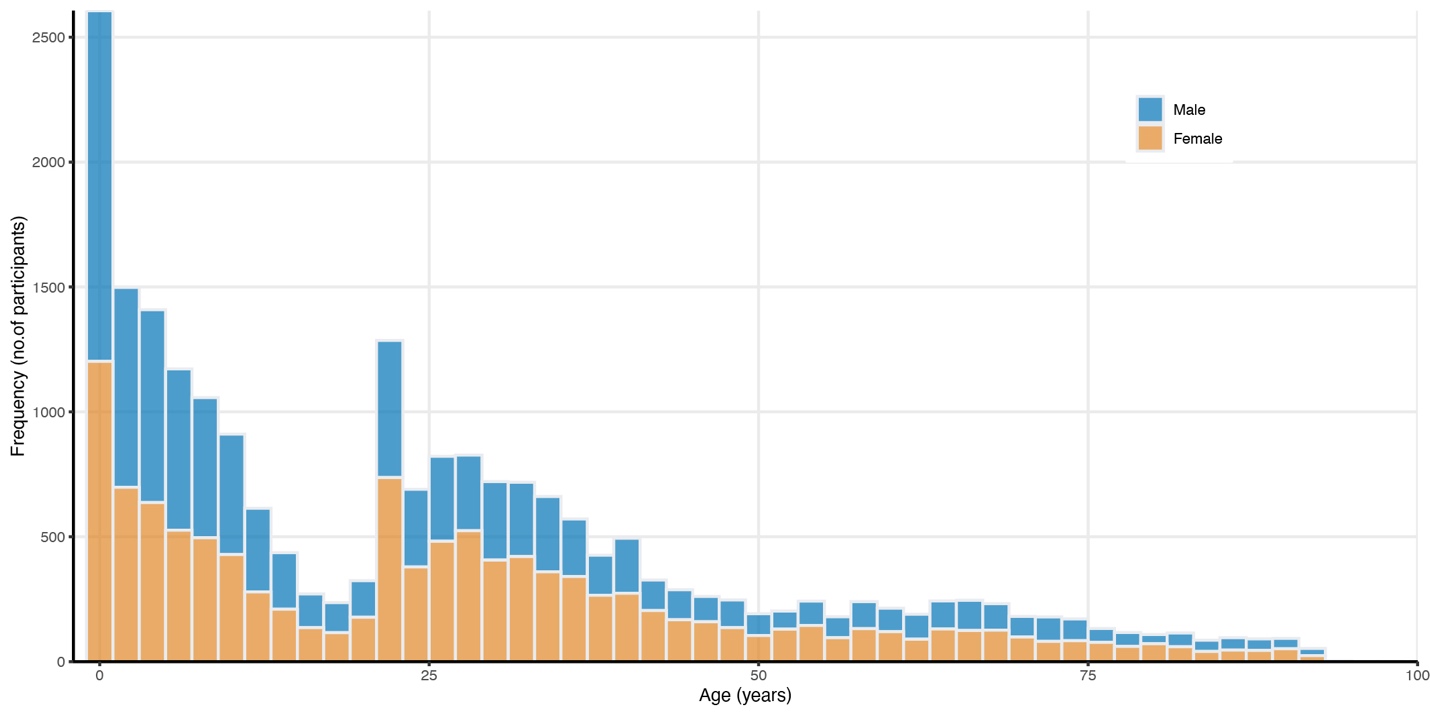


The bar plot shows number of participants of different genders in EMRs in the implementation dataset after putting the triage system into application.
