# Supplementary figures and images for: Evolution of the “Internet Plus Health Care” Mode Enabled by Artificial Intelligence: Development and Application of an Outpatient Triage System
Source: J Med Internet Res. 2024 Oct 30;26:e51711. doi: 10.2196/51711 (PMC11561436; doi:10.2196/51711)

## Multimedia Appendix 8

## Time Trend of System Users


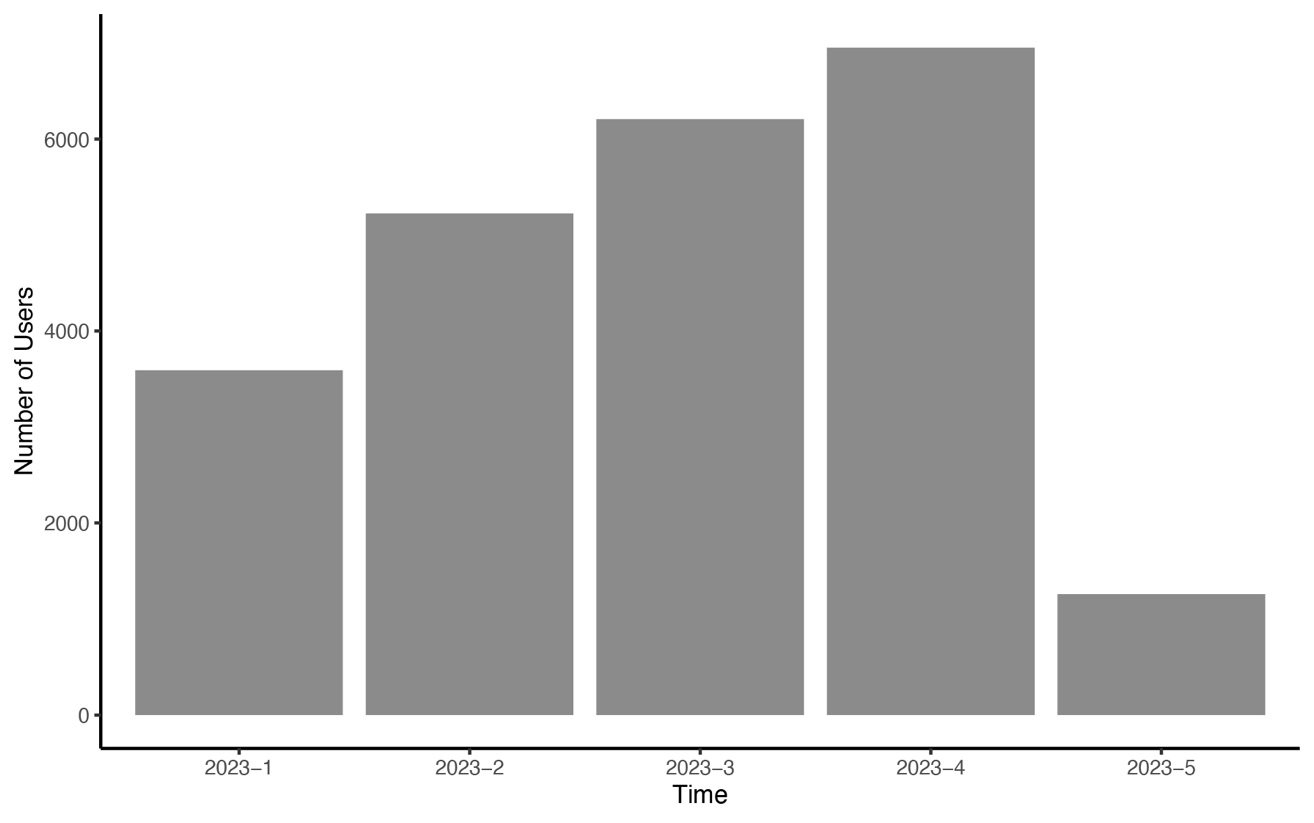

Supplement: Multimedia Appendix 8 [file jmir_v26i1e51711_app8.docx]
