# Supplementary material for: Evolution of the “Internet Plus Health Care” Mode Enabled by Artificial Intelligence: Development and Application of an Outpatient Triage System
Source: J Med Internet Res. 2024 Oct 30;26:e51711. doi: 10.2196/51711 (PMC11561436; doi:10.2196/51711)
Supplement: Multimedia Appendix 9 [file jmir_v26i1e51711_app9.docx]

**Here is the main source code of the triage system, please refer to the dependencies in github:** [**https://github.com/genggui001/triage**](https://github.com/genggui001/triage)

import json

import datetime,time

import os

import shutil

os.environ["CUDA_VISIBLE_DEVICES"] = '0'

# os.environ["TF_KERAS"] = '1'

# os.environ["RECOMPUTE"] = '1'

seed = 123456

import tensorflow as tf

# tf.compat.v1.disable_eager_execution()

import json

import numpy as np

from bert4keras.backend import keras

# from bert4keras.backend import multilabel_categorical_crossentropy

# from bert4keras.layers import GlobalPointer

from bert4keras.snippets import sequence_padding, DataGenerator

from bert4keras.backend import K

from bert4keras.tokenizers import Tokenizer, load_vocab

from bert4keras.models import build_transformer_model

from bert4keras.optimizers import Adam

from bert4keras.optimizers import extend_with_gradient_accumulation, extend_with_weight_decay, extend_with_piecewise_linear_lr

from keras.models import Model

from tqdm.notebook import tqdm

# 模型路径

config_path = './pretrain_weights/chinese_roformer-char_L-12_H-768_A-12/bert_config.json'

checkpoint_path = './pretrain_weights/chinese_roformer-char_L-12_H-768_A-12/bert_model.ckpt'

dict_path = './pretrain_weights/chinese_roformer-char_L-12_H-768_A-12/vocab.txt'

# 建立分词器

tokenizer = Tokenizer(dict_path, do_lower_case=True)

maxlen = 256

epochs = 45

batch_size = 32

# steps_per_epoch = 800

learning_rate = 2e-5

import pickle

with open("./data/categorical_data/train_data.pkl", "rb") as f:

train_data = pickle.load(f)

with open("./data/categorical_data/dev_data.pkl", "rb") as f:

dev_data = pickle.load(f)

print(len(train_data))

print(len(dev_data))

with open("./data/categorical_data/level_1_oc_list.json", "r") as f:

level_1_oc_list = json.load(f)

level_1_oc_map = {item: idx for idx, item in enumerate(level_1_oc_list)}

print(len(level_1_oc_list))

print(len(level_1_oc_map))

with open("./data/categorical_data/diease_list.json", "r") as f:

diseases_list = json.load(f)

diseases_map = {item: idx for idx, item in enumerate(diseases_list)}

print(len(diseases_list))

print(len(diseases_map))

def log_softmax(x, axis=-1):

tmp_max = np.max(x, axis=axis,keepdims=True)

x = x - tmp_max

logsumexp = np.log(np.sum(np.exp(x),axis=axis,keepdims=True))

return x - logsumexp

dd_symptom_text_map = {

# "腹痛": "肚子痛",

}

def get_symptoms_text(mr_data_item):

text = ""

if "症状" in mr_data_item:

symptoms = mr_data_item['症状']

for symptom in symptoms:

symptom_text = symptom['症状名称']

if "程度" in symptom:

symptom_text = symptom['程度'] + symptom_text

if "是否存在" in symptom and symptom['是否存在'] == '不存在':

symptom_text = '无' + symptom_text

if "发生时段" in symptom:

symptom_text = symptom_text + symptom['发生时段']

if "性质" in symptom:

symptom_text = symptom_text + symptom['性质']

if "持续时间" in symptom:

symptom_text = symptom_text + '、主要发生在' + symptom['持续时间']

if "颜色" in symptom:

symptom_text = symptom_text + "、颜色呈" + symptom['颜色']

if "频率" in symptom:

symptom_text = symptom_text + "、频率为" + symptom['频率']

if "诱因" in symptom:

symptom_text = symptom_text + "、" + symptom['诱因']

if "部位" in symptom:

tmp_bodyparts = []

for bodypart in symptom['部位']:

if bodypart not in symptom_text:

tmp_bodyparts.append(bodypart)

if len(tmp_bodyparts) > 0:

symptom_text = "、".join(symptom['部位']) + symptom_text

# 规则模板

symptom_text = dd_symptom_text_map.get(symptom_text, symptom_text)

text += (symptom_text + "，")

return text

def add_disease_info(disease, disease_text):

if "类型" in disease:

disease_text = disease['类型'] + disease_text

if "程度" in disease:

disease_text = disease['程度'] + disease_text

if "开始时间" in disease:

disease_text = disease_text + '、开始于' + disease['开始时间']

if "结束时间" in disease:

disease_text = disease_text + '、结束于' + disease['结束时间']

if "持续时间" in disease:

disease_text = disease_text + '、持续' + disease['持续时间']

return disease_text

def get_now_exist_diseases_text(mr_data_item):

text = ""

if "疾病" in mr_data_item:

now_exist_diseases = [

disease

for disease in mr_data_item['疾病']

if disease['发生时间'] == '现在' and disease['是否存在'] != '不存在'

]

if len(now_exist_diseases) > 0:

text = (text + "患有")

for disease in now_exist_diseases:

disease_text = disease['疾病名称']

disease_text = add_disease_info(disease, disease_text)

text += (disease_text + "、")

return text

def get_now_not_exist_diseases_text(mr_data_item):

text = ""

if "疾病" in mr_data_item:

not_exist_diseases = [

disease

for disease in mr_data_item['疾病']

if disease['发生时间'] == '现在' and disease['是否存在'] == '不存在'

]

if len(not_exist_diseases) > 0:

text = (text + "否认")

for disease in not_exist_diseases:

disease_text = disease['疾病名称']

disease_text = add_disease_info(disease, disease_text)

text += (disease_text + "、")

return text

def get_now_past_diseases_text(mr_data_item):

text = ""

if "疾病" in mr_data_item:

# 既往史

past_diseases = [

disease

for disease in mr_data_item['疾病']

if disease['发生时间'] == '既往' and disease['是否存在'] != '不存在'

]

if len(past_diseases) > 0:

# 分割既往史

text = (text + "存在")

for disease in past_diseases:

disease_text = (disease['疾病名称'] + "史")

disease_text = add_disease_info(disease, disease_text)

text += (disease_text + "、")

return text

def get_now_not_past_diseases_text(mr_data_item):

text = ""

if "疾病" in mr_data_item:

# 既往史

not_past_diseases = [

disease

for disease in mr_data_item['疾病']

if disease['发生时间'] == '既往' and disease['是否存在'] == '不存在'

]

if len(not_past_diseases) > 0:

text = (text + "否认")

for disease in not_past_diseases:

disease_text = (disease['疾病名称'] + "史")

disease_text = add_disease_info(disease, disease_text)

text += (disease_text + "、")

return text

def gen_age_sex(mr_data_item):

age = 30

sex = None

# 基本信息解析

if "病人信息" in mr_data_item:

basic_info = mr_data_item["病人信息"]

if "年龄" in basic_info:

age = float(basic_info['年龄'] / 31536000000.0)

if "性别" in basic_info and basic_info['性别'] in {"男","女"}:

sex = basic_info['性别']

if age <= 0.13150684931506848:

age_text = "新生患儿"

elif age < 14:

age_text = "患儿"

elif age > 60:

age_text = "老年患者"

else:

age_text = "患者"

return age_text, sex

def get_inspection_text(mr_data_item):

text = ""

if "检查史" in mr_data_item and len(mr_data_item['检查史']) > 0:

text = (text + "已行")

for inspection in mr_data_item['检查史']:

inspection_text = inspection['检查名称']

text += (inspection_text + "、")

text = text[:-1] + "检查。"

return text

def get_drug_text(mr_data_item):

text = ""

if "用药史" in mr_data_item and len(mr_data_item['用药史']) > 0:

text = (text + "曾使用")

for drug in mr_data_item['用药史']:

drug_text = drug['用药名称']

text += (drug_text + "、")

text = text[:-1] + "药物。"

return text

def get_surgery_text(mr_data_item):

text = ""

if "治疗史" in mr_data_item and len(mr_data_item['治疗史']) > 0:

text = (text + "曾行")

for surgery in mr_data_item['治疗史']:

surgery_text = surgery['治疗名称']

text += (surgery_text + "、")

text = text[:-1] + "。"

return text

def gen_mr_text(mr_data_item):

text = ""

age_text, sex = gen_age_sex(mr_data_item)

text += (age_text + "，")

if sex is not None:

text += (sex + "，")

text += get_symptoms_text(mr_data_item)

text += get_now_exist_diseases_text(mr_data_item)

text += get_now_not_exist_diseases_text(mr_data_item)

inspection_text = get_inspection_text(mr_data_item)

if len(inspection_text) > 0:

text = text[:-1] + "。" + inspection_text

surgery_text = get_surgery_text(mr_data_item)

if len(surgery_text) > 0:

text = text[:-1] + "。" + surgery_text

drug_text = get_drug_text(mr_data_item)

if len(drug_text) > 0:

text = text[:-1] + "。" + drug_text

past_text = get_now_past_diseases_text(mr_data_item)

past_text += get_now_not_past_diseases_text(mr_data_item)

if len(past_text) > 0:

text = text[:-1] + "。" + past_text

text = text[:-1] + "。"

return text

import copy

np.random.seed(123456)

def get_random_sample_data(

data,

prob,

keep_one=False

):

if len(data) == 0:

return []

real_choice_count = len(data) * prob

real_choice_count = np.random.poisson(lam=real_choice_count)

real_choice_count = min(real_choice_count, len(data))

if keep_one:

real_choice_count = max(real_choice_count, 1)

c_idxs = np.random.choice(list(range(len(data))), size=real_choice_count, replace=False)

return [data[c_idx] for c_idx in c_idxs]

def get_random_mr_data(

data_item,

exist_symptom_prob = 0.5,

not_exist_symptom_prob = 0.2,

exist_disease_prob = 0.3,

not_exist_disease_prob = 0.1,

exist_inspection_prob = 0.1,

exist_drug_prob = 0.1,

exist_surgery_prob = 0.1,

):

new_data_item = copy.deepcopy(data_item)

# z症状

exist_symptoms = get_random_sample_data([

(idx, item)

for idx, item in enumerate(new_data_item['症状'])

if item['是否存在'] == '存在'

], prob=exist_symptom_prob, keep_one=True)

not_exist_symptoms = get_random_sample_data([

(idx, item)

for idx, item in enumerate(new_data_item['症状'])

if item['是否存在'] == '不存在'

], prob=not_exist_symptom_prob)

new_data_item['症状'] = [

item

for idx, item in sorted(exist_symptoms + not_exist_symptoms, key=lambda x:x[0])

]

# 疾病

exist_diseases = get_random_sample_data([

(idx, item)

for idx, item in enumerate(new_data_item['疾病'])

if item['是否存在'] == '存在'

], prob=exist_disease_prob)

not_exist_diseases = get_random_sample_data([

(idx, item)

for idx, item in enumerate(new_data_item['疾病'])

if item['是否存在'] == '不存在'

], prob=not_exist_disease_prob)

new_data_item['疾病'] = [

item

for idx, item in sorted(exist_diseases + not_exist_diseases, key=lambda x:x[0])

]

# 检查史

new_data_item['检查史'] = [

tmp

for _, tmp in sorted(get_random_sample_data([

(idx, item)

for idx, item in enumerate(new_data_item['检查史'])

], prob=exist_inspection_prob), key=lambda x:x[0])

]

# 用药史

new_data_item['用药史'] = [

tmp

for _, tmp in sorted(get_random_sample_data([

(idx, item)

for idx, item in enumerate(new_data_item['用药史'])

], prob=exist_drug_prob), key=lambda x:x[0])

]

# 治疗史

new_data_item['治疗史'] = [

tmp

for _, tmp in sorted(get_random_sample_data([

(idx, item)

for idx, item in enumerate(new_data_item['治疗史'])

], prob=exist_surgery_prob), key=lambda x:x[0])

]

return new_data_item

train_data_flatten = []

disease_log_prob = np.zeros((len(diseases_list),), dtype=np.float32)

oc_log_prob = np.zeros((len(level_1_oc_list),), dtype=np.float32)

for item in tqdm(train_data):

for disease in item['diseases']:

train_data_flatten.append({

'text': item['text'],

'mc_data': item['mc_data'],

'level_1_oc': item['level_1_oc'],

'disease': disease,

})

disease_log_prob[diseases_map[disease]] += 1

oc_log_prob[level_1_oc_map[item['level_1_oc']]] += 1

disease_log_prob = log_softmax(disease_log_prob)

oc_log_prob = log_softmax(oc_log_prob)

len(train_data_flatten)

class data_generator(DataGenerator):

"""数据生成器

"""

def __iter__(self, random=False):

batch_token_ids, batch_segment_ids = [], []

batch_disease_labels = []

batch_oc_labels = []

for is_end, (data_item) in self.sample(random):

# 进行一次随机采样

text = gen_mr_text(get_random_mr_data(data_item['mc_data']))

token_ids, segment_ids = tokenizer.encode(text, maxlen=maxlen)

batch_token_ids.append(token_ids)

batch_segment_ids.append(segment_ids)

oc_label = level_1_oc_map[data_item['level_1_oc']]

batch_oc_labels.append([oc_label])

disease_label = diseases_map[data_item['disease']]

batch_disease_labels.append([disease_label])

if len(batch_token_ids) == self.batch_size or is_end:

batch_token_ids = sequence_padding(batch_token_ids)

batch_segment_ids = sequence_padding(batch_segment_ids)

batch_disease_labels = sequence_padding(batch_disease_labels)

batch_oc_labels = sequence_padding(batch_oc_labels)

yield {

"Input-Token": batch_token_ids,

"Input-Segment": batch_segment_ids,

}, {

"disease_label": batch_disease_labels,

"oc_label": batch_oc_labels,

}

batch_token_ids, batch_segment_ids = [], []

batch_disease_labels = []

batch_oc_labels = []

train_generator = data_generator(train_data_flatten, batch_size)

steps_per_epoch = len(train_generator)

base = build_transformer_model(

config_path,

checkpoint_path,

model='roformer',

# keep_tokens=keep_tokens,

return_keras_model=False,

)

output = keras.layers.Lambda(lambda x: x[:, 0])(base.model.output)

disease_label_output = keras.layers.Dense(

units=len(diseases_list),

activation='linear',

kernel_initializer=base.initializer,

name="disease_label",

)(output)

oc_label_output = keras.layers.Dense(

units=len(level_1_oc_list),

activation='linear',

kernel_initializer=base.initializer,

name="oc_label",

)(output)

model = keras.models.Model(

base.model.input,

[disease_label_output, oc_label_output]

)

def get_optimizer(

learning_rate,

num_warmup_steps,

num_train_steps,

weight_decay_rate=0.01,

exclude_from_weight_decay=['Norm', 'bias'],

grad_accum_steps=1,

):

optimizer = extend_with_weight_decay(Adam)

optimizer = extend_with_piecewise_linear_lr(optimizer)

optimizer_params = {

'learning_rate': learning_rate,

'lr_schedule': {

num_warmup_steps * grad_accum_steps: 1.0,

num_train_steps * grad_accum_steps: 0.0,

},

'weight_decay_rate': weight_decay_rate,

'exclude_from_weight_decay': exclude_from_weight_decay,

}

if grad_accum_steps > 1:

optimizer = extend_with_gradient_accumulation(optimizer, name='AdamWG')

optimizer_params['grad_accum_steps'] = grad_accum_steps

return optimizer(**optimizer_params)

optimizer=get_optimizer(

learning_rate=learning_rate,

num_warmup_steps=0,

num_train_steps=steps_per_epoch*epochs,

)

def make_sparse_categorical_focal_loss(

gamma,

# class_weight=None,

from_logits=False,

):

def sparse_categorical_focal_loss(

y_true, y_pred,

):

# Process focusing parameter

tgamma = tf.convert_to_tensor(gamma, dtype=tf.dtypes.float32)

# gamma_rank = gamma.shape.rank

# scalar_gamma = gamma_rank == 0

# # Process class weight

# if class_weight is not None:

# class_weight = tf.convert_to_tensor(class_weight,

# dtype=tf.dtypes.float32)

y_true = tf.dtypes.cast(y_true, dtype=tf.dtypes.int32)

labels = tf.reshape(y_true, [-1])

y_true = tf.reshape(y_true, [-1, 1])

if from_logits:

logits = y_pred

probs = tf.nn.softmax(y_pred, axis=-1)

else:

probs = y_pred

logits = tf.math.log(tf.clip_by_value(y_pred, _EPSILON, 1 - _EPSILON))

xent_loss = tf.nn.sparse_softmax_cross_entropy_with_logits(

labels=labels,

logits=logits,

)

# print(probs, y_true)

probs = tf.gather(probs, y_true, batch_dims=1)

probs = tf.reshape(probs, [-1])

# print(probs)

# if not scalar_gamma:

# gamma = tf.gather(gamma, y_true, axis=0, batch_dims=y_true_rank)

focal_modulation = (1 - probs) ** tgamma

loss = focal_modulation * xent_loss

# if class_weight is not None:

# class_weight = tf.gather(class_weight, y_true, axis=0,

# batch_dims=y_true_rank-1)

# loss *= class_weight

# if reshape_needed:

# loss = tf.reshape(loss, y_pred_shape[:-1])

return loss

return sparse_categorical_focal_loss

import functools

model.compile(

loss={

"disease_label": make_sparse_categorical_focal_loss(

gamma=2,

from_logits=True,

),

"oc_label": make_sparse_categorical_focal_loss(

gamma=2,

from_logits=True,

),

},

# loss={

# "disease_label": "sparse_categorical_crossentropy",

# "oc_label": "sparse_categorical_crossentropy",

# },

optimizer=optimizer,

)

def largest_indices(flat, n):

"""Returns the n largest indices from a numpy array.

Arguments:

array {np.ndarray} -- data array

n {int} -- number of elements to select

Returns:

tuple[np.ndarray, np.ndarray] -- tuple of ndarray

each ndarray is index

"""

# flat = array.flatten()

# flat = array

indices = np.argpartition(flat, -n)[-n:]

indices = indices[np.argsort(-flat[indices])]

return indices

def predict(

data,

random_mr_data=False

):

batch_token_ids, batch_segment_ids = [], []

for data_item in data:

if random_mr_data is True:

text = gen_mr_text(get_random_mr_data(data_item['mc_data']))

else:

text = gen_mr_text(data_item['mc_data'])

token_ids, segment_ids = tokenizer.encode(text, maxlen=maxlen)

batch_token_ids.append(token_ids)

batch_segment_ids.append(segment_ids)

batch_token_ids = sequence_padding(batch_token_ids)

batch_segment_ids = sequence_padding(batch_segment_ids)

pre_disease_label_scores, pre_oc_label_scores = model.predict([

batch_token_ids, batch_segment_ids,

], batch_size=128, verbose=1)

# pre_disease_label_scores = pre_disease_label_scores

# pre_oc_label_scores = pre_oc_label_scores

# pre_oc_labels = np.argmax(pre_oc_label_scores, axis=-1)

# pre_oc_labels = [level_1_oc_list[item] for item in pre_oc_labels]

pre_oc_label_top20 = []

for pre_oc_label_score in pre_oc_label_scores:

label_top20 = largest_indices(

pre_oc_label_score,

n=20

)

label_top20 = [level_1_oc_list[item] for item in label_top20]

pre_oc_label_top20.append(label_top20)

# pre_disease_labels = []

pre_disease_label_top20 = []

for pre_disease_label_score in pre_disease_label_scores:

# pre_disease_label = np.where(pre_disease_label_score > 0)[0]

# pre_disease_label = [diseases_list[item] for item in pre_disease_label]

# pre_disease_labels.append(pre_disease_label)

label_top20 = largest_indices(

pre_disease_label_score,

n=20

)

label_top20 = [diseases_list[item] for item in label_top20]

pre_disease_label_top20.append(label_top20)

return pre_oc_label_top20, pre_disease_label_top20

def eval_oc_topk(pre_oc_label_top20s, true_oc_labels):

top1_acc = 0.0

top3_acc = 0.0

top5_acc = 0.0

top10_acc = 0.0

top20_acc = 0.0

for pre_oc_label_top20, true_oc_label in zip(

pre_oc_label_top20s,

true_oc_labels

):

pre_oc_label_top1 = set(pre_oc_label_top20[:1])

pre_oc_label_top3 = set(pre_oc_label_top20[:3])

pre_oc_label_top5 = set(pre_oc_label_top20[:5])

pre_oc_label_top10 = set(pre_oc_label_top20[:10])

pre_oc_label_top20 = set(pre_oc_label_top20)

true_oc_label = set(true_oc_label)

if true_oc_label.issubset(pre_oc_label_top1):

top1_acc += 1

if true_oc_label.issubset(pre_oc_label_top3):

top3_acc += 1

if true_oc_label.issubset(pre_oc_label_top5):

top5_acc += 1

if true_oc_label.issubset(pre_oc_label_top10):

top10_acc += 1

if true_oc_label.issubset(pre_oc_label_top20):

top20_acc += 1

top1_acc /= len(true_oc_labels)

top3_acc /= len(true_oc_labels)

top5_acc /= len(true_oc_labels)

top10_acc /= len(true_oc_labels)

top20_acc /= len(true_oc_labels)

print('oc top1_acc: %.5f, top3_acc: %.5f, oc top5_acc: %.5f, top10_acc: %.5f, top20_acc: %.5f' %

(top1_acc, top3_acc, top5_acc, top10_acc, top20_acc))

return top1_acc, top3_acc, top5_acc, top10_acc, top20_acc

def eval_disease_topk(pre_disease_label_top20s, true_disease_labels):

top3_acc = 0.0

top5_acc = 0.0

top10_acc = 0.0

top20_acc = 0.0

for pre_disease_label_top20, true_disease_label in zip(

pre_disease_label_top20s,

true_disease_labels

):

pre_disease_label_top3 = set(pre_disease_label_top20[:3])

pre_disease_label_top5 = set(pre_disease_label_top20[:5])

pre_disease_label_top10 = set(pre_disease_label_top20[:10])

pre_disease_label_top20 = set(pre_disease_label_top20)

true_disease_label = set(true_disease_label)

if true_disease_label.issubset(pre_disease_label_top3):

top3_acc += 1

if true_disease_label.issubset(pre_disease_label_top5):

top5_acc += 1

if true_disease_label.issubset(pre_disease_label_top10):

top10_acc += 1

if true_disease_label.issubset(pre_disease_label_top20):

top20_acc += 1

top3_acc /= len(true_disease_labels)

top5_acc /= len(true_disease_labels)

top10_acc /= len(true_disease_labels)

top20_acc /= len(true_disease_labels)

print('disease top3_acc: %.5f, disease top5_acc: %.5f, top10_acc: %.5f, top20_acc: %.5f' %

(top3_acc, top5_acc, top10_acc, top20_acc))

return top3_acc, top5_acc, top10_acc, top20_acc

# from sklearn.metrics import accuracy_score

def cal_eval(random_mr_data=False):

dev_pre_oc_label_top20, dev_pre_disease_label_top20 = predict(dev_data, random_mr_data=random_mr_data)

dev_true_oc_labels = [[item['level_1_oc']] for item in dev_data]

dev_oc_acc, _, _, _, _ = eval_oc_topk(dev_pre_oc_label_top20, dev_true_oc_labels)

dev_true_disease_labels = []

for data_item in dev_data:

dev_true_disease_label = []

for disease in data_item['diseases']:

if disease in diseases_map:

dev_true_disease_label.append(disease)

dev_true_disease_labels.append(dev_true_disease_label)

# dev_disease_f1 = eval_disease_f1(dev_pre_disease_labels, dev_true_disease_labels)

dev_disease_top3_acc, _, _, _ = eval_disease_topk(dev_pre_disease_label_top20, dev_true_disease_labels)

dev_score = dev_oc_acc + dev_disease_top3_acc

return dev_score

from keras.callbacks import Callback, CSVLogger

class SaveModelBestCheckpoint(Callback):

"""自动保存最佳模型

"""

def __init__(self, model_saved_path):

self.model_saved_path = model_saved_path

if not os.path.isdir(self.model_saved_path):

os.makedirs(self.model_saved_path)

self.best_score = None

def on_epoch_end(self, epoch, logs=None):

print("-------------Ordinary-data------------------")

tmp_ordinary_score = cal_eval(random_mr_data=False)

print("-------------Random-data------------------")

tmp_random_score = cal_eval(random_mr_data=True)

tmp_score = tmp_ordinary_score + tmp_random_score

if self.best_score is None or tmp_score > self.best_score:

self.best_score = tmp_score

self.model.save(os.path.join(self.model_saved_path, "best_weights.h5"), overwrite=True)

class SaveModelLastCheckpoint(Callback):

"""自动保存最新模型

"""

def __init__(self, model_saved_path):

self.model_saved_path = model_saved_path

if not os.path.isdir(self.model_saved_path):

os.makedirs(self.model_saved_path)

def on_epoch_end(self, epoch, logs=None):

self.model.save(os.path.join(self.model_saved_path, "last_weights.h5"), overwrite=True)

projectName = "roformer 新华病历直接预测-focal_loss多分类-病历构造"

print(projectName + ' Train...')

now = time.strftime("%Y-%m-%d_%H-%M-%S")

projectPath = './param/outputModelWeights/{}'.format(projectName)

if not os.path.isdir(projectPath): os.makedirs(projectPath)

resultPath = projectPath + '/{}/'.format(now)

os.makedirs(resultPath)

print(resultPath)

callbacks = [

SaveModelBestCheckpoint(resultPath),

SaveModelLastCheckpoint(resultPath),

CSVLogger(resultPath + 'training.log'),

]

model.fit(

train_generator.forfit(),

epochs=epochs,

steps_per_epoch=steps_per_epoch,

callbacks=callbacks,

)

model.load_weights(resultPath + "best_weights.h5")

cal_eval(random_mr_data=True)

cal_eval(random_mr_data=False)
